# Investigation of an ^18^F-labelled Imidazopyridotriazine for Molecular Imaging of Cyclic Nucleotide Phosphodiesterase 2A

**DOI:** 10.3390/molecules23030556

**Published:** 2018-03-02

**Authors:** Susann Schröder, Barbara Wenzel, Winnie Deuther-Conrad, Rodrigo Teodoro, Mathias Kranz, Matthias Scheunemann, Ute Egerland, Norbert Höfgen, Detlef Briel, Jörg Steinbach, Peter Brust

**Affiliations:** 1 Department of Neuroradiopharmaceuticals, Institute of Radiopharmaceutical Cancer Research, Helmholtz-Zentrum Dresden-Rossendorf, Leipzig 04318, Germany; b.wenzel@hzdr.de (B.W.); w.deuther-conrad@hzdr.de (W.D.-C.); r.teodoro@hzdr.de (R.T.); m.kranz@hzdr.de (M.K.); m.scheunemann@hzdr.de (M.S.); j.steinbach@hzdr.de (J.S.); p.brust@hzdr.de (P.B.); 2 BioCrea GmbH, Radebeul 01445, Germany; ute.egerland@outlook.de (U.E.); norbert.hoefgen@dynabind.com (N.H.); 3 Pharmaceutical/Medicinal Chemistry, Institute of Pharmacy, Faculty of Medicine, Leipzig University, Leipzig 04103, Germany; briel@uni-leipzig.de

**Keywords:** Phosphodiesterase 2A (PDE2A), secondary messengers, PDE2A radioligands, positron emission tomography (PET), neuroimaging, metabolic stability, micellar liquid chromatography (MLC)

## Abstract

Specific radioligands for in vivo visualization and quantification of cyclic nucleotide phosphodiesterase 2A (PDE2A) by positron emission tomography (PET) are increasingly gaining interest in brain research. Herein we describe the synthesis, the ^18^F-labelling as well as the biological evaluation of our latest PDE2A (radio-)ligand 9-(5-Butoxy-2-fluorophenyl)-2-(2-([^18^F])fluoroethoxy)-7-methylimidazo[5,1-*c*]pyrido[2,3-*e*][1,2,4]triazine (([^18^F])**TA5**). It is the most potent PDE2A ligand out of our series of imidazopyridotriazine-based derivatives so far (IC_50_ hPDE2A = 3.0 nM; IC_50_ hPDE10A > 1000 nM). Radiolabelling was performed in a one-step procedure starting from the corresponding tosylate precursor. In vitro autoradiography on rat and pig brain slices displayed a homogenous and non-specific binding of the radioligand. Investigation of stability in vivo by reversed-phase HPLC (RP-HPLC) and micellar liquid chromatography (MLC) analyses of plasma and brain samples obtained from mice revealed a high fraction of one main radiometabolite. Hence, we concluded that [^18^F]**TA5** is not appropriate for molecular imaging of PDE2A neither in vitro nor in vivo. Our ongoing work is focusing on further structurally modified compounds with enhanced metabolic stability.

## 1. Introduction

The dual-substrate specific enzyme cyclic nucleotide phosphodiesterase 2A (PDE2A) degrades the secondary messengers cyclic adenosine monophosphate (cAMP) as well as cyclic guanosine monophosphate (cGMP) and thus, considerably affects the signaling cascades of these cyclic nucleotides by altering their intracellular levels [1,2,3]. The PDE2A protein is mainly expressed in the brain and predominantly in structures of the limbic system such as cortex, hippocampus, striatum, substantia nigra, globus pallidus, habenulae, bulbus olfactorius, tuberculum olfactorium, and amygdala [4,5]. This specific localization indicates a regulatory role of PDE2A in important neuronal processes associated to learning, memory and emotion [2,3]. Therefore, PDE2A is suggested to be involved in the pathophysiology of neurodegenerative and neuropsychiatric disorders like Alzheimer´s disease and depression [2,3,5,6].

Pharmacological inhibition of PDE2A activity has been proven to enhance neuronal plasticity due to increased intracellular levels of cAMP and cGMP [2,7,8,9,10,11]. This effect is considered as a highly promising approach in drug development regarding treatment of related neurological diseases [2,6,7,8,9,12]. However, the complex relationship between PDE2A activity and pathological changes in the brain is not entirely understood so far [2].

Accordingly, specific radioligands for in vivo imaging and quantification of PDE2A in the brain by positron emission tomography (PET) have been gaining importance during the last years [13]. Besides the lack of brain-penetrating radiometabolites, the most significant criterion for an appropriate PDE2A radioligand is a high selectivity versus the PDE10A protein due to the comparable distribution pattern of both enzymes in the brain [14].

**Figure 1:**
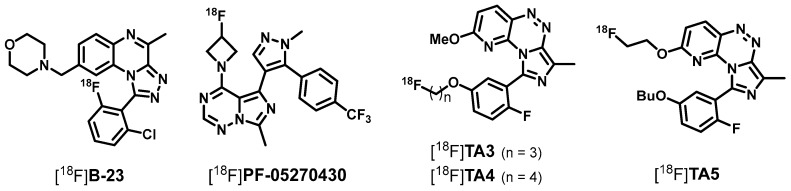
PDE2A radioligands developed by Janssen ([^18^F]**B-23** [6,15]), Pfizer ([^18^F]**PF-05270430** [6,16]), and our group ([^18^F]**TA3**–**5** [13,19,20]).

The first two PDE2A radioligands have been published in 2013 by Janssen Pharmaceutica NV (Beerse, Belgium), [^18^F]**B-23** [6,15], and Pfizer Inc., (New York, NY, USA) [^18^F]**PF-05270430** [6,16] (Figure 1). In biodistribution and microPET imaging studies in rats, [^18^F]**B-23** showed a high uptake in the striatum [15]. However, due to the low PDE2A/PDE10A selectivity of this radioligand (IC_50_ hPDE2A = 1 nM; IC_50_ rPDE10A = 11 nM) and the detection of radiometabolites in the brain (at 2 min post injection (p.i.): 4%; at 10 min p.i.: 18% of total activity) [15], [^18^F]**B-23** is not recommended to be suitable for molecular imaging of the PDE2A protein. The highly potent PDE2A radioligand [^18^F]**PF-05270430** (IC_50_ hPDE2A = 0.5 nM; IC_50_ hPDE10A > 3000 nM) has been evaluated preclinically in monkeys [16] and already in a clinical PET study in humans [17,18]. The promising results stated so far, such as PDE2A-specific accumulation with highest uptake in putamen, caudate and nucleus accumbens, a good metabolic stability (intact radioligand at 120 min p.i. in plasma: 40% of total activity) and a favorable kinetic profile [18], point out that [^18^F]**PF-05270430** is an appropriate radioligand for PET imaging of PDE2A in the human brain.

Recently, the development of three further PDE2A radioligands, [^18^F]**TA3**, [^18^F]**TA4** and [^18^F]**TA5** (TA stands for Triazine) (Figure 1), has been reported by our group [13,19,20,21].

For ([^18^F])**TA3** and ([^18^F])**TA4**, the optimized (radio-)syntheses, the in vitro characterization as well as the biological evaluation in mice have been described previously [19]. Briefly, these two radioligands are suitable for imaging of the PDE2A protein in vitro as demonstrated by the region-specific and displaceable binding in autoradiographic studies on rat brain slices. However, [^18^F]**TA3** and [^18^F]**TA4** undergo a fast metabolic degradation in mice with a high fraction of polar radiometabolites in the brain (at 30 min p.i.: >70% of total activity). It is supposed that these radiometabolites are formed by cytochrome P450 (CYP450) enzyme-induced cleavage of the ^18^F-bearing alkoxyphenyl side chains resulting in the corresponding brain-penetrating ^18^F-alkyl alcohols, aldehydes or carboxylic acids [22,23]. Consequently, [^18^F]**TA3** and [^18^F]**TA4** are not applicable for PET neuroimaging of PDE2A [19].

It should be noted that radiotracers bearing a [^18^F]fluoroalkoxyphenyl group do not per se undergo a metabolic *O*-dealkylation. For example, the radioligand [^18^F]**FET** (*O*-(2-[^18^F]fluoroethyl)-L-tyrosine) for PET imaging of brain tumors [24,25,26] as well as the *O*-(2-[^18^F]fluoromethyl) and the *O*-(2-[^18^F]fluoropropyl) derivatives ([^18^F]**FMT** and [^18^F]**FPT**) demonstrated high in vivo stability (intact [^18^F]**FET**, [^18^F]**FMT**, and [^18^F]**FPT** at 60 min p.i. in mouse plasma: > 90% of total activity [27]; intact [^18^F]**FET** at 60 min p.i. in human plasma: >90% [24,28]). Furthermore, the PDE10A radioligand [^18^F]**MNI-659** (2-(2-(3-(4-(2-[^18^F]Fluoroethoxy)phenyl)-7-methyl-4-oxo-3,4-dihydrochinazolin-2-yl)ethyl)-4-isopropoxyisoindolin-1,3-di-on) [29,30,31] showed a high and region-specific accumulation in the human brain [29,31] although it is of moderate metabolic stability (intact [^18^F]**MNI-659** at 120 min p.i. in human plasma: 20% of total activity [29]). Thus, it is suggested that no brain-penetrating radiometabolites are formed indicating [^18^F]**MNI-659** does also not undergo a CYP450-induced cleavage of the [^18^F]fluoroethoxyphenyl side chain. For those reasons, we did not exclude the fluoroalkoxy moiety in order to develop PDE2A ligands with enhanced in vivo stability compared to [^18^F]**TA3** and [^18^F]**TA4**. Instead, we intended to reach this purpose by changing the position of the fluoroalkoxy group from the phenolic side chain in **TA3** and **TA4** to the pyridinyl moiety. Regarding that, it has been described for diacylethylenediamine derivatives as diacylglycerol aycyltransferase-1 inhibitors that replacement of an ethoxybenzoyl group with a less lipophilic 2-ethoxypyridinyl or 2-(2,2,2-trifluoroethoxy)pyridinyl moiety revealed a significant increasing in vitro metabolic stability in mouse, rat and human hepatic microsomal preparations [32]. 

Finally, our efforts led to the novel PDE2A (radio-)ligand ([^18^F])**TA5** (Figure 1) as shortly mentioned in former publications [13,20]. Herein, we report on the synthetic route, the ^18^F-labelling, and the in vitro and in vivo characterization of ([^18^F])**TA5** in detail.

## 2. Results and Discussion

### 2.1. Organic Syntheses and Inhibitory Potency

**Scheme 1:**
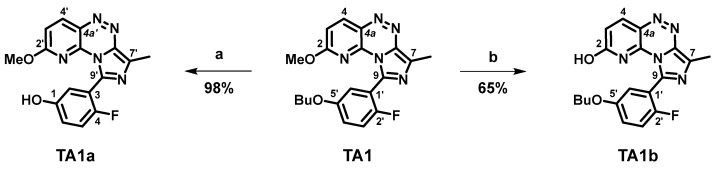
Syntheses of the 1-phenol **TA1a** [19] and the 2-pyridinol **TA1b**. Reagents and Conditions: (a) 3.05 eq. BBr_3_ (1 M in CH_2_Cl_2_), CH_2_Cl_2_, ≤5 °C, 2 h; (b) 1 eq. K_2_CO_3_∙1.5H_2_O, 1.5 eq. NH(CH_3_)_2_, DMSO/H_2_O (2.5:1, *v*/*v*), 100 °C, 5 h and room temperature (RT) overnight.

The synthesis of our selected lead compound **TA1** comprises five steps [7], which have already been optimized [19]. Starting from **TA1** we established appropriate *O*-dealkylation procedures to selectively split the alkoxy groups either (**a**) at the 5´-phenol moiety [19] or (**b**) at the 2-pyridine function (Scheme 1).

The usage of boron tribromide for the cleavage of aromatic alkylethers is a very common and well-known method [33,34,35,36,37]. However and to the best of our knowledge, the regioselective *O*-dealkylation of an phenolic ether with boron tribromide in the presence of a 2-methoxy pyridine has been described only once before [38]. This approach led to the easy accessibility of our 5´-fluoroalkoxy derivatives (**TA2**–**4**) as well as the corresponding tosylate precursors for ^18^F-labelling [19]. Notably, the 2-methoxy function in **TA1** generally showed a very high stability against acidic reagents (e.g., up to 10 eq. BBr_3_ [19] or conc. HCl under reflux). Due to these experiences, we supposed that the cleavage of the 2-methoxy group requires basic conditions instead of an acidic strategy. This assumption was confirmed by the reaction of **TA1** with dimethylamine as nitrogenous nucleophile in the presence of potassium carbonate and the formation of the desired 2-pyridinol **TA1b** (Scheme 1). Remarkably, similar demethylation reactions at 2-methoxy pyridines have been reported by using sodium thiolates [39,40] but with the herein described method, one could forego the need of a sulfurous nucleophile that brings some advantages in the laboratory work.

**Scheme 2:**
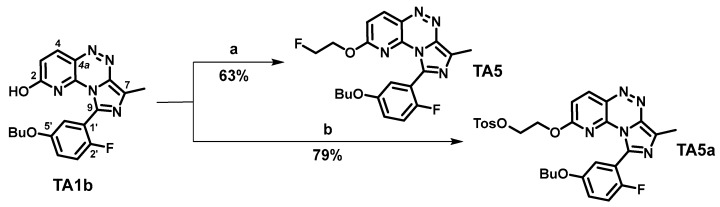
Syntheses of the novel 2-fluoroethoxy PDE2A ligand **TA5** and the tosylate precursor **TA5a**. Reagents and Conditions: (a) 1.5 eq. F-(CH_2_)_2_-I, 3 eq. K_2_CO_3_∙1.5H_2_O, MeCN, 70–80 °C, 5 h and room temperature (RT) overnight; (b) 2 eq. TosO-(CH_2_)_2_-OTos, 4 eq. K_2_CO_3_∙1.5H_2_O, MeCN, 60–70 °C, 5 h and RT overnight.

Finally, the novel 2-fluoroethoxy derivative **TA5** [13,20] was successfully synthesized with 63% yield using the 2-pyridinol **TA1b** and fluoroethyl iodide as fluoroalkylating agent (Scheme 2).

The 2-pyridinol **TA1b** exists in equilibrium with its cyclic amide as 2-pyridone or 2-lactam that is suggested to be the more stable form in both the solid state and in solution [41,42,43]. It has been described that reactions of metal salts of 2-pyridones with alkyl halides often result in *N*- and *O*-alkylated product mixtures depending on the nature of the metal cation, the substitution pattern at the pyridone ring, the molecular structure of the alkyl halide and the solvent used [44,45,46]. In the herein reported synthesis, only the formation of the preferred *O*-fluoroethylated compound **TA5** was observed, as confirmed by two-dimensional NMR spectroscopy (see Supplementary Materials). Therefore, it is assumed that the nitrogen of the 2-pyridone in **TA5** is sterically hindered against the electrophilic attack of the fluoroethyl iodide by the adjacent imidazotriazine moiety.

Evaluation of the novel 2-fluoroethoxy derivative **TA5** in an enzyme assay [7] resulted in a slightly higher affinity towards the human PDE2A protein as compared with the lead compound **TA1** (Table 1). Above all, **TA5** showed a considerably increased PDE2A/PDE10A selectivity compared to **TA1** as well as the former developed 5´-fluoroalkoxy derivatives **TA2**–**4** and thus, **TA5** is the most potent PDE2A ligand out of this series so far [13,19,20].

This tendency points out that the substitution patterns at both the 5**′**-phenol and the 2-pyridine position in **TA1** play a decisive role for the selectivity versus PDE10A, which is needed for PET neuroimaging of PDE2A due to the similar distribution of both enzymes in the brain [14]. The herein observed strong impact of the chain length of the 5**′**-fluoroalkoxy group in **TA2**–**4** on the PDE2A affinity and mainly the PDE2A/PDE10A selectivity is in accordance to the effect seen for the previously reported imidazotriazine-based compounds where the selectivity versus PDE10A increases in the following order: 5**′**-methoxy < 5**′**-ethoxy < 5**′**-propoxy < 5**′**-butoxy with PDE10A/PDE2A selectivity ratios of 3, 4, 40, and >120, respectively [7]. Notably, in this series the 2-pyridine was permanently substituted by a methoxy function [7]. 

Accordingly, **TA5** was selected for ^18^F-labelling and the corresponding tosylate precursor **TA5a** was synthesized by reaction of 2-pyridinol **TA1b** with ethane-1,2-diyl bis(4-methylbenzenesulfonate) in 79% yield (see Scheme 2).

### 2.2. Radiosynthesis, In Vitro Stability and Lipophilicity

**Scheme 3:**
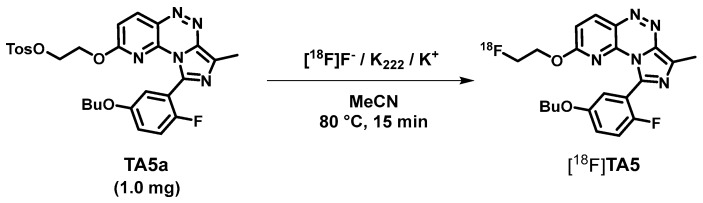
One-step nucleophilic ^18^F-labelling procedure to generate the novel PDE2A radioligand [^18^F]**TA5**.

Based on our experiences in the radiosyntheses of [^18^F]**TA3** and [^18^F]**TA4** [19], the optimized parameters for the one-step ^18^F-labelling procedure have been adopted to generate the novel PDE2A radioligand [^18^F]**TA5** (Scheme 3). Nucleophilic substitution of the tosylate group of the precursor **TA5a** with the anhydrous K^+^/[^18^F]F^−^/K_222_-carbonate complex in acetonitrile resulted in [^18^F]**TA5** with a high radiochemical yield of 65.3 ± 2.1% (n = 3; based on radio-TLC analysis of the crude product). Stability of the precursor under the reaction conditions over 20 min was proven by HPLC and no ^18^F-labelled by-product was detected.

**Figure 2:**
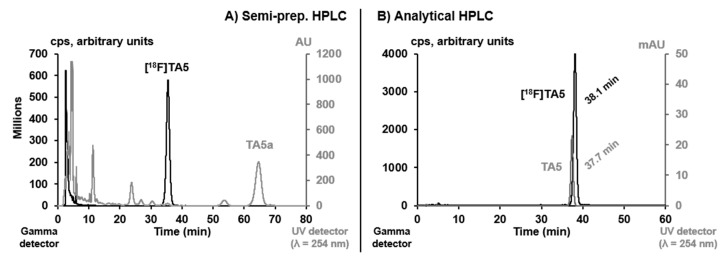
(**A**) Semi-preparative HPLC profile of the crude reaction mixture for isolation of [^18^F]**TA5** (column: Reprosil-Pur C18-AQ, 250 × 10 mm, particle size: 10 µm; eluent: 50% MeCN/20 mM NH_4_OAc_aq._; flow: 5 mL/min); (**B**) analytical HPLC profile of the formulated radioligand [^18^F]**TA5** spiked with the non-radioactive reference compound **TA5** (column: Reprosil-Pur C18-AQ, 250 × 4.6 mm, particle size: 5 µm; eluent: 52% MeCN/20 mM NH_4_OAc_aq._; flow: 1 mL/min).

Isolation of [^18^F]**TA5** was performed by semi-preparative HPLC (*t_R_* = 34–38 min, see Figure 2) followed by purification and concentration via solid-phase extraction on a pre-conditioned reversed-phase (RP) cartridge and elution with absolute ethanol. After evaporation of the solvent at 70 °C, the radioligand was finally formulated in sterile isotonic saline with a maximum ethanol content of 10% (*v*/*v*) for better solubility. The identity of [^18^F]**TA5** was confirmed by analytical HPLC using an aliquot of the final product spiked with the non-radioactive reference compound **TA5** (Figure 2). 

The novel PDE2A radioligand [^18^F]**TA5** was synthesized with an overall radiochemical yield of 44.8 ± 5.7% (n = 3), a molar activity of 47.0 ± 7.6 GBq/µmol (n = 3, end of synthesis (EOS)) and a high radiochemical purity of ≥99%.

In vitro stability of [^18^F]**TA5** was proven in phosphate-buffered saline (PBS, pH 7.4), *n*-octanol and pig plasma. Samples of each medium were analyzed by radio-TLC and radio-HPLC after 1 h incubation at 37 °C and no degradation or defluorination of the radioligand has been observed.

The distribution coefficient of [^18^F]**TA5** was determined by partitioning between *n*-octanol and phosphate-buffered saline (PBS, pH 7.4) at ambient temperature using the conventional shake-flask method. The obtained logD value of 2.52 ± 0.23 (n = 4) indicates a lipophilicity of [^18^F]**TA5** which should allow moderate passive diffusion at the blood-brain barrier. However, we observed a strong discrepancy between the experimentally determined logD value and the calculated distribution coefficients (ChemBioDraw Ultra 12.0 (CambridgeSoft Corporation, Cambridge, MA, USA): clogP = 5.26; ACD/Labs 12.0: clogD_7.4_ = 4.78). The experimentally determined higher hydrophilicity of [^18^F]**TA5** could be a result of solvation effects associated with formation of hydrogen bonds or ionization of the radioligand in the buffered aqueous system. These effects may be underestimated in the software-based determination and thus, the calculated values of lipophilicity are often higher than those obtained experimentally [47].

Remarkably, this has also been observed for [^18^F]**TA4** while for [^18^F]**TA3** the logD values obtained from the shake-flask method correlated well with the calculated data. Compared to [^18^F]**TA5**, the experimentally determined logD values of [^18^F]**TA3** (3.57 [19]) and [^18^F]**TA4** (2.99 [19]) are higher indicating that [^18^F]**TA5** is the least lipophilic derivative in this series. In contrast, the calculated data show the reverse tendency. Regarding the molecular structures of the herein discussed PDE2A ligands, we expect that the lipophilicity increases in the order **TA3** < **TA4** < **TA5** due to the additional methylene groups in **TA4** and **TA5**, respectively, which corresponds with the calculated tendency. This assumption is supported by HPLC where **TA3** elutes at the shortest retention time followed by **TA4** and **TA5** (gradient and isocratic mode). A probable conclusion could be: the more lipophilic a compound is, the higher is the difference between the logD values obtained from the shake-flask method and the calculated data for lipophilicity. Nevertheless, this postulation needs to be confirmed.

### 2.3. Biological Evaluation—In Vitro Autoradiography and In Vivo Metabolism

For autoradiographic studies, sagittal slices of rat and pig brain were incubated with [^18^F]**TA5**. Non-specific binding was assessed via co-incubation with an access of either **TA1** or **TA5**. However, the activity pattern on both, rat and pig brain sections, showed a homogenous and non-displaceable distribution which indicates insufficient specificity of [^18^F]**TA5** under in vitro conditions. This is in contrast to the demonstrated suitability of [^18^F]**TA3** and [^18^F]**TA4** for in vitro imaging of PDE2A [19]. Compared to these radioligands, the lipophilicity of [^18^F]**TA5** is suggested to be higher as indicated by the elution order in the HPLC analyses and the calculated distribution coefficients. This would result in an increased plasma protein binding leading to a reduced availability of free [^18^F]**TA5**. However, up to now we have no reasonable explanation for the observed high non-specific binding of [^18^F]**TA5** in vitro. Notably, with availability of [^18^F]**TA5** the in vitro and in vivo investigations have been performed in parallel.

**Figure 3:**
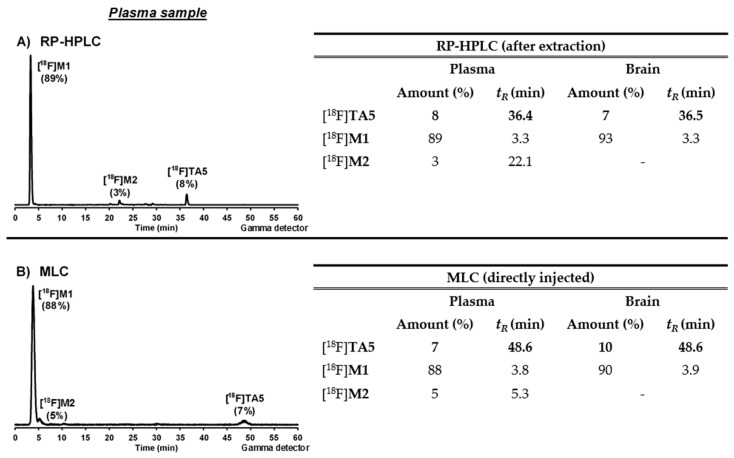
Representative in vivo metabolism study of mouse plasma and brain samples at 30 min p.i. of [^18^F]**TA5** (~70 MBq): (**A**) RP-HPLC chromatogram of extracted plasma sample (column: Reprosil-Pur C18-AQ, 250 x 4.6 mm, particle size: 5 µm; gradient: 10–90–10% MeCN/20 mM NH_4_OAc_aq._; flow: 1 mL/min); (**B**) MLC chromatogram of directly injected plasma sample (column: Reprosil-Pur C18-AQ, 250 x 4.6 mm, particle size: 10 µm; gradient: 3-30-3% 1-PrOH/100 mM SDS_aq._, 10 mM Na_2_HPO_4aq._; flow: 1 mL/min); Tables: percentages and retention times of intact [^18^F]**TA5** and radiometabolites from RP-HPLC analysis after extraction and MLC analysis of directly injected samples.

Encouraged by our experiences in the metabolism studies with [^18^F]**TA3** and [^18^F]**TA4** [19] regarding reliable qualitative and quantitative data, radiometabolites of [^18^F]**TA5** and parent compound were analyzed by (i) reversed-phase HPLC (RP-HPLC) after conventional extraction and (ii) micellar liquid chromatography (MLC [48]). Blood plasma and brain homogenate samples were obtained from CD-1 mice at 30 min post injection of ~70 MBq of the radioligand. In both, plasma and brain, only 7–10% of total activity were representing non-metabolized [^18^F]**TA5** (see Figure 3). A high fraction of a main radiometabolite [^18^F]**M1** (~90%) was detected in RP-HPLC and MLC eluting at a very short retention time of 3–4 min indicating a high hydrophilicity. 

The MLC method was recently established in our group for rapid analysis of radiometabolites [19,49]. Briefly, plasma and homogenized brain samples were dissolved in aqueous sodium dodecyl sulphate (SDS), as an important part of the MLC eluent, and injected directly into the MLC system. There is no further work-up needed and thus, there is no loss of activity prior analysis of the samples. Hence, it is possible to quantify the real composition of total activity in the injected biological material. In contrast, the twofold extraction procedure is work-intensive and time-consuming and most important, the extractability of highly polar radiometabolites from denatured proteins might be low resulting in only a partial recovery of total activity. With the herein applied protocol we previously observed that for [^18^F]fluoride [19]. Consequently, the amount of intact radioligand would be overestimated if radiometabolites with an ionic character are formed leading to misinterpretation of the data.

The percentages of intact [^18^F]**TA5** achieved from the RP-HPLC analysis of the extracted samples fit well with those from samples directly analyzed by MLC (see Figure 3). This is not surprising, because with the conventional extraction procedure high recoveries of ≥93% of total activity were observed. The chromatograms obtained with both methods differ only regarding the elution profile. While in the RP-HPLC radiometabolite [^18^F]**M2** elutes after 22 min, in the MLC it elutes already after 5 min (Figure 3). This might be a result of the various retention mechanisms in these two systems. Therefore, analysis of biological samples with RP-HPLC as well as with MLC is beneficial to reliably characterize the metabolic profile of a newly developed radioligand.

The highly polar main radiometabolite [^18^F]**M1** was detected in both, plasma and brain samples, pointing out that [^18^F]**M1** may cross the blood-brain barrier. Regarding the identity of [^18^F]**M1**, it is presumed that no defluorination or formation of any ionic radiometabolite of [^18^F]**TA5** occurs due to the fact that ionic compounds are not completely extractable from biological material with the method used here. Accordingly, [^18^F]**M1** is suggested to be 2-[^18^F]fluoroethanol resulting from a cytochrome P450 enzyme-induced metabolic degradation of the ^18^F-fluoroethoxy side chain in [^18^F]**TA5**. This assumption is further supported by the fact that 2-[^18^F]fluoroethanol and the oxidized 2-[^18^F]fluoroacetaldehyde or 2-[^18^F]fluoroacetate are able to enter the brain [50,51,52,53]. To clarify whether there is an in vivo defluorination of [^18^F]**TA5**, PET imaging or biodistribution investigations could provide more information regarding accumulation of activity in the bones. However, due to the low stability of [^18^F]**TA5** in mice and formation of brain-penetrating radiometabolites we decided to abstain from further in vivo studies with this radioligand. In conclusion, the rate of metabolic degradation could not be reduced by moving the [^18^F]fluoroalkoxy group from the phenolic position in [^18^F]**TA3** and [^18^F]**TA4** to the pyridinyl moiety in [^18^F]**TA5**. 

Finally, with the novel highly potent PDE2A ligand **TA5** we have shown that changes in the substitution pattern at the pyridinyl moiety of our imidazopyridotriazine lead compound **TA1** can significantly increase the PDE2A/PDE10A selectivity. Starting from the 2-pyridone **TA1b** it is possible to introduce different substituents at the 2-pyridinyl position with or without an alkoxy linker. Thus, our current work is focused on further structurally modified derivatives with feasibly enhanced in vivo stability at the 2-pyridinyl side chain, for example with branched fluoroalkoxy or fluoroalkyl groups as well as cyclic and aromatic fluorine-bearing functions [54,55,56]. 

## 3. Materials and Methods

### 3.1. General Information

Chemicals were purchased from standard commercial sources in analytical grade and were used without further purification. Radio-/TLCs were performed on pre-coated silica gel plates (Alugram^®^ Xtra SIL G/UV_254_; Polygram^®^ SIL G/UV_254_, Roth, Karlsruhe, Germany). The compounds were localized at 254 nm (UV lamp) and/or by staining with aqueous KMnO_4_ solution or ninhydrin solution. Radio-TLC was recorded using a bioimaging analyzer system (BAS-1800 II, Fuji Photo Film, Co. Ltd., Tokyo, Japan) and images were evaluated with Aida 2.31 software (raytest Isotopenmessgeräte GmbH, Straubenhardt, Germany). Column chromatography was conducted on silica gel (0.06–0.20 mm, Roth). HPLC separations were performed on JASCO systems equipped with UV detectors from JASCO and activity detectors from raytest Isotopenmessgeräte GmbH (GABI Star, Straubenhardt, Germany).

Semi-preparative HPLC conditions were: Column: Reprosil-Pur C18-AQ, 250 × 10 mm, particle size: 10 µm; eluent: 50% MeCN/20 mM NH_4_OAc_aq._; flow: 5 mL/min; ambient temperature; UV detection at 254 nm.

Analytical HPLC conditions were: Column: Reprosil-Pur C18-AQ, 250 × 4.6 mm, particle size: 5 µm; gradient: 0–10 min: 10% MeCN, 10–35 min: 10% → 90% MeCN, 35–45 min: 90% MeCN, 45–50 min: 90% → 10% MeCN, 50–60 min: 10% MeCN/20 mM NH_4_OAc_aq._; isocratic: 52% MeCN/20 mM NH_4_OAc_aq._; flow: 1 mL/min; ambient temperature; UV detection at 254 nm. Molar activity was determined on the base of a calibration curve (0.2–20 µg **TA5**) carried out under isocratic HPLC conditions (52% MeCN/20 mM NH_4_OAc_aq._) using chromatograms obtained at 270 nm as the maximum of UV absorbance.

MLC conditions were: Column: Reprosil-Pur C18-AQ, 250 × 4.6 mm, particle size: 10 µm; gradient: 0–15 min: 3% 1-PrOH, 15–40 min: 3% → 30% 1-PrOH; 40–49 min: 30% 1-PrOH, 49–50 min: 30% → 3% 1-PrOH; 50–60 min: 3% 1-PrOH/100 mM SDS, 10 mM Na_2_HPO_4_; flow: 1 mL/min; ambient temperature; UV detection at 254 nm. Notably, a pre-column with 10 mm length was used and frequently exchanged to expand the lifetime of the RP-column.

NMR spectra (^1^H, ^13^C, ^19^F) were recorded on Mercury 300/Mercury 400 (Varian, Palo Alto, CA, USA) or Fourier 300/Avance DRX 400 Bruker (Billerica, MA, USA) instruments. The hydrogenated residue of deuteriated solvents and/or tetramethylsilane (TMS) were used as internal standards for ^1^H-NMR (CDCl_3_, δ_H_ = 7.26; DMSO-*d_6_*, δ_H_ = 2.50) and ^13^C-NMR (CDCl_3_, δ_C_ = 77.2; DMSO-*d_6_*, δ_C_ = 39.5). The chemical shifts (δ) are reported in ppm (s, singlet; d, doublet; t, triplet; q, quartet; p, pentet (quintet); h, hexett (sextet); m, multiplet) and the related coupling constants (*J*) are reported in Hz. High resolution mass spectra (ESI +/−) were recorded on an Impact II^TM^ instrument (Bruker Daltonics).

No-carrier-added (n.c.a.) [^18^F]fluoride (*t*_1/2_ = 109.8 min) was produced via the [^18^O(p,n)^18^F] nuclear reaction by irradiation of [^18^O]H_2_O (Hyox 18 enriched water, Rotem Industries Ltd, Arava, Israel) on a Cyclone^®^18/9 (iba RadioPharma Solutions, Louvain-la-Neuve, Belgium) with fixed energy proton beam using Nirta^®^ [^18^F]fluoride XL target.

### 3.2. Organic Syntheses

The optimized syntheses of the lead compound **TA1** and the 1-phenol intermediate **TA1a** are published previously [19]. All final compounds described in this manuscript meet the purity requirements determined by HPLC, NMR and HR-MS.

#### 3.2.1. 9-(5-Butoxy-2-fluorophenyl)-7-methylimidazo[5,1-*c*]pyrido[2,3-*e*][1,2,4]triazin-2-ol (**TA1b**)

Compound **TA1** (0.50 g, 1 eq.) was dissolved in dimethyl sulfoxide and water (14 mL, 2.5:1, *v*/*v*) followed by addition of K_2_CO_3_∙1.5H_2_O (0.22 g, 1 eq.) and dimethylamine (250 µL, 1.5 eq.). The reaction mixture was stirred at 100 °C for 5 h and at ambient temperature overnight. After evaporation of the solvent, the residue was dissolved in CH_2_Cl_2_ (10 mL) and washed once with aq. saturated solutions of NaHCO_3_ and NaCl, and water (5 mL each). The aqueous phase was extracted with CH_2_Cl_2_ (5 mL). The combined organic phases were dried over Na_2_SO_4_ and filtered. Evaporation of the solvent and subsequent purification by column chromatography (EtOAc/CH_2_Cl_2_, 1:4 to 100% EtOAc, *v*/*v*) afforded a yellow solid of **TA1b** (0.31 g, 65%). ^1^H-NMR (400 MHz, CDCl_3_): δ_H_ = 0.88 (t, *J* = 7.4, 3H, O(CH_2_)_3_CH_3_); 1.31 (h-like, *J* = 7.3, 2H, O(CH_2_)_2_CH_2_CH_3_); 1.52–1.63 (m, 2H, OCH_2_CH_2_CH_2_CH_3_); 2.81 (s, 3H, 7-C-CH_3_); 3.66 (t, *J* = 6.6, 2H, OCH_2_(CH_2_)_2_CH_3_); 6.35 (dt, *J* = 9.0, 3.6, 1H_Ar_, 4′-H); 6.64 (t, *J* = 9.0, 1H_Ar_, 3′-H); 6.95 (d, *J* = 8.8, 1H_Ar_, 3-H); 7.07 (dd, *J* = 5.6, 3.1, 1H_Ar_, 6′-H); 8.54 (d, *J* = 8.8, 1H_Ar_, 4-H); 11.08 (br s, 1H, 2-C-OH). ^13^C-NMR (75 MHz, CDCl_3_): δ_C_ = 12.4 (s, 1C_prim_, 7-C-CH_3_); 13.9 (s, 1C_prim_, O(CH_2_)_3_CH_3_); 19.2 (s, 1C_sec_, O(CH_2_)_2_CH_2_CH_3_); 31.2 (s, 1C_sec_, OCH_2_CH_2_CH_2_CH_3_); 68.4 (s, 1C_sec_, OCH_2_(CH_2_)_2_CH_3_); 112.1 (s, 1C_ArH_, 3-C); 115.6 (d, *J* = 23.2, 1C_ArH_, 3′-C); 115.9 (d, *J* = 2.3, 1C_ArH_, 6′-C); 117.2 (d, *J* = 8.0, 1C_ArH_, 4′-C); 119.7 (d, *J* = 16.3, 1C_Ar_, 1′-C); 128.2 (s, 1C_Ar_, 4a-C); 132.6 (s, 1C_Ar_, 9-C); 134.9 (s, 1C_Ar_, 10a-C); 137.6 (s, 1C_Ar_, 7-C); 139.2 (s, 1C_Ar_, 6a-C); 140.9 (s, 1C_ArH_, 4-C); 154.8 (d, *J* = 1.9, 1C_Ar_, 5′-C); 155.1 (d, overlap, *J* = 243.2, 1C_Ar_, 2′-C); 164.9 (s, 1C_Ar_, 2-C). ^19^F-NMR (377 MHz, CDCl_3_): δ_F_ = −119.95 (p, *J* = 5.0, 1F_Ar_, 2′-C-F).

#### 3.2.2. 9-(5-Butoxy-2-fluorophenyl)-2-(2-fluoroethoxy)-7-methylimidazo[5,1-*c*]pyrido[2,3-*e*]-[1,2,4]triazine (**TA5**)

To a solution of compound **TA1b** (0.31 g, 1 eq.) in MeCN (25 mL), K_2_CO_3_∙1.5H_2_O (0.42 g, 3 eq.) and 1-fluoro-2-iodoethane (104 µL, 1.5 eq.) were added. The yellow suspension was stirred at 70–80 °C for 5 h and at ambient temperature overnight. The mixture was filtered and the solvent was evaporated. The residue was dissolved in CH_2_Cl_2_ (10 mL), washed with water (5 mL) and then with citric acid (5 mL, 25%). The aqueous phase was extracted with CH_2_Cl_2_ (2 mL). The combined organic phases were dried over Na_2_SO_4_ and filtered. After evaporation of the solvent the crude product was purified by column chromatography (EtOAc/CH_2_Cl_2_, 1:4 to 100% EtOAc, *v*/*v*) yielding a yellow solid of **TA5** (0.22 g, 63%). ^1^H-NMR (400 MHz, DMSO-*d*_6_): δ_H_ = 0.90 (t, *J* = 7.4, 3H, O(CH_2_)_3_CH_3_); 1.41 (h-like, *J* = 7.4, 2H, O(CH_2_)_2_CH_2_CH_3_); 1.67 (p-like, *J* = 6.6, 2H, OCH_2_CH_2_CH_2_CH_3_); 2.80 (s, 3H, 7C-CH_3_); 3.98 (t, distorted, *J* = 6.5, 3H, OCH_2_(CH_2_)_2_CH_3_, OCHH′-CH_2_F); 4.04 (t, poorly resolved, partly overlapped A part of AA′BB′X, *J* = 3.9, 1H, OCHH′-CH_2_F); 4.47 (dt, poorly resolved, *J* = 47.7, 4.0, B part of AA′BB′X, 2H, OCH_2_-CH_2_F); 7.08–7.16 (m, 1H_Ar_, 4′-H); 7.17–7.25 (m, 2H_Ar_, 4′-H, 3-H); 7.31 (t, *J* = 9.2, 1H_Ar_, 3′-H); 8.71 (d, *J* = 8.8, 1H_Ar_, 4-H). ^13^C-NMR (75 MHz, DMSO-*d_6_*): δ_C_ = 12.4 (s, 1C_prim_, 7-C-CH_3_); 13.6 (s, 1C_prim_, O(CH_2_)_3_CH_3_); 18.7 (s, 1C_sec_, O(CH_2_)_2_CH_2_CH_3_); 30.7 (s, 1C_sec_, OCH_2_CH_2_CH_2_CH_3_); 66.1 (d, *J* = 19.1, 1C_sec_, OCH_2_CH_2_F); 68.0 (s, 1C_sec_, OCH_2_(CH_2_)_2_CH_3_); 81.1 (d, *J* = 167.0, 1C_sec_, OCH_2_CH_2_F); 112.2 (s, 1C_ArH_, 3-C); 116.0 (d, *J* = 22.9, 1C_ArH_, 3′-C); 117.4 (d, *J* = 8.4, 1C_ArH_, 4′-C); 117.5 (d, *J* = 1.7, 1C_ArH_, 6′-C); 120.3 (d, *J* = 16.6, 1C_Ar_, 1′-C); 128.0 (s, 1C_Ar_, 4a-C); 131.6 (s, 1C_Ar_, 9-C); 133.4 (s, 1C_Ar_, 10a-C); 136.7 (s, 1C_Ar_, 7-C); 138.9 (s, 1C_Ar_, 6a-C); 141.1 (s, 1C_ArH_, 4-C); 154.3 (d, *J* = 2.2, 1C_Ar_, 5′-C); 154.6 (d, overlapping, *J* = 239.9, 1C_Ar_, 2′-C); 162.9 (s, 1C_Ar_, 2-C). ^19^F-NMR (282 MHz, DMSO-*d_6_*): δ_F_ = −122.53 (dt, *J* = 9.5, 4.7, 1F_Ar_, 2′-F); −223.50 (tt, *J* = 47.8, 29.7, 1F, O(CH_2_)_2_F). HR-MS (ESI) *m*/*z*: calcd. for [C_21_H_22_F_2_N_5_O_2_]^+^ = 414.1736; found = 414.1739 [M + H]^+^.

#### 3.2.3. 2-((9-(5-Butoxy-2-fluorophenyl)-7-methylimidazo[5,1-*c*]pyrido[2,3-*e*][1,2,4]-triazin-2-yl)oxy)ethyl-4-methylbenzenesulfonate (**TA5a**)

Compound **TA1b** (0.85 g, 1 eq.) was dissolved in MeCN (20 mL) and K_2_CO_3_∙1.5 H_2_O (0.15 g, 4 eq.) and ethane-1,2-diyl bis(4-methylbenzenesulfonate) (0.17 g, 2 eq.; synthesized according to the literature [19,57]) were added. After stirring at 60–70 °C for 5 h and at ambient temperature overnight, the yellow suspension was filtered followed by evaporation of the solvent. The residue was dissolved in CH_2_Cl_2_ (10 mL), washed once with water and citric acid (25%, 5 mL each) and then the aqueous phase was extracted with CH_2_Cl_2_ (2 mL). The combined organic phases were dried over Na_2_SO_4_, filtered and the solvent was evaporated. Purification by column chromatography (EtOAc/CH_2_Cl_2_, 1:5, *v*/*v*) afforded a yellow solid of **TA5a** (0.10 g, 79%). ^1^H-NMR (400 MHz, DMSO-*d_6_*): δ_H_ = 0.92 (t, *J* = 7.4, 3H, O(CH_2_)_3_CH_3_); 1.37–1.48 (m, 2H, O(CH_2_)_2_CH_2_CH_3_); 1.63–1.77 (m, 2H, OCH_2_CH_2_CH_2_CH_3_); 2.15 (s, 3H, 4″-C-CH_3_); 2.83 (s, 3H, 7-C-CH_3_); 3.87–3.92 (m, 2H, OCH_2_CH_2_OTs); 3.97–4.03 (m, 4H, OCH_2_CH_2_OTs, OCH_2_(CH_2_)_2_CH_3_); 7.03 (d, *J* = 8.8, 1H_Ar_, 3-H); 7.10 (ddd, *J* = 9.0, 4.0, 3.1, 1H_Ar_, 4′-H); 7.15 (dd, *J* = 8.6, 0.8, 2H_Ar_, 3″-H, 5″-H); 7.19–7.29 (m, 2H_Ar_, 3′-H, 6′-H); 7.56 (d, *J* = 8.3, 2H_Ar_, 2″-H, 6″-H); 8.69 (d, *J* = 8.8, 1H_Ar_, 4-H). ^13^C-NMR (75 MHz, DMSO-*d*_6_): δ_C_ = 12.4 (s, 1C_prim_, 7-C-CH_3_); 13.7 (s, 1C_prim_, O(CH_2_)_3_CH_3_); 18.7 (s, 1C_sec_, O(CH_2_)_2_CH_2_CH_3_); 20.8 (s, 1C_prim_, 4″-C-CH_3_); 30.7 (s, 1C_sec_, OCH_2_CH_2_CH_2_CH_3_); 64.5 (s, 1C_sec_, OCH_2_CH_2_OTs); 67.3 (s, 1C_sec_, OCH_2_CH_2_OTs); 68.1 (s, 1C_sec_, OCH_2_(CH_2_)_2_CH_3_); 112.3 (s, 1C_ArH_, 3-C); 116.0 (d, *J* = 22.9, 1C_ArH_, 3′-C); 117.4 (d, *J* = 8.1, 1C_ArH_, 4′-C); 117.5 (d, *J* = 1.9, 1C_ArH_, 6′-C); 120.1 (d, *J* = 16.6, 1C_Ar_, 1′-C); 127.5 (s, 2C_ArH_, 2″-C, 6″-C); 127.9 (s, 1C_Ar_, 4a-C); 129.7 (s, 2C_ArH_, 3″-C, 5″-C); 131.5 (s, 1C_Ar_, 9-C); 131.6 (s, 1C_Ar_, 1″-C); 133.1 (s, 1C_Ar_, 10a-C); 136.9 (s, 1C_Ar_, 7-C); 138.9 (s, 1C_Ar_, 6a-C); 140.9 (s, 1C_ArH_, 4-C); 144.7 (s, 1C_Ar_, 4″-C); 154.45 (d, *J* = 1.9, 1C_Ar_, 5′-C); 154.49 (d, overlap, *J* = 240.2, 1C_Ar_, 2′-C); 162.5 (s, 1C_Ar_, 2-C). ^19^F-NMR (282 MHz, DMSO-*d*_6_): δ_F_ = −122.41 (dt, *J* = 9.5, 4.9, 1F_Ar_, 2′-F). HR-MS (ESI) *m*/*z*: calcd. for [C_28_H_29_FN_5_O_5_S]^+^ = 566.1867; found = 566.1868 [M + H]^+^.

### 3.3. In Vitro Affinity Assay

The inhibitory potencies of **TA1**–**5** for human recombinant PDE2A and PDE10A proteins were determined by BioCrea GmbH (Radebeul, Germany) [7].

### 3.4. Radiochemistry

The aqueous solution of no-carrier-added [^18^F]fluoride (1–2 GBq) was trapped on a Chromafix^®^ 30 PS-HCO_3_^−^ cartridge (MACHEREY-NAGEL GmbH & Co. KG, Düren, Germany). The activity was eluted with 300 µL of an aqueous K_2_CO_3_-solution (1.78 mg, 12.9 µmol) into a 4 mL V-vial containing Kryptofix 2.2.2 (K_222_, 11.2 mg, 29.7 µmol) in 1 mL MeCN. The K^+^/[^18^F]F^−^/K_222_-carbonate complex was azeotropically dried under vacuum and nitrogen flow within 7–10 min using a Discover PETwave Microwave CEM^®^ (75 W, 50–60 °C, power cycling mode). Two aliquots of MeCN (2 × 1.0 mL) were added during the drying procedure and the final complex was dissolved in 500 µL MeCN ready for radiolabelling.

The aliphatic radiolabelling of the tosylate **TA5a** (1 mg in 500 µL MeCN) was performed under conventional heating at 80 °C for 15 min. Aliquots of the reaction mixture were analyzed by radio-TLC (EtOAc/DCM, 1:1, *v*/*v*) to determine the radiochemical yield of the crude product. After dilution with water (1:1, *v*/*v*), the crude reaction mixture was applied to an isocratic semi-preparative HPLC (see General Information) for isolation of the desired radioligand [^18^F]**TA5** (*t_R_* = 34–38 min). The collected fractions were diluted with water (total volume: 40 mL), passed through a Sep-Pak^®^ C18 Plus light cartridge (Waters, Milford, MA, USA; pre-conditioned with 20 mL of absolute EtOH and 60 mL water), and eluted with 0.75 mL of absolute EtOH. Evaporation of the solvent at 70 °C under a gentle nitrogen stream and subsequent formulation of the radioligand in sterile isotonic saline containing 10% EtOH (*v*/*v*) afforded a [^18^F]**TA5**-solution usable for biological investigations.

The identity of the radioligand was proved by analytical radio-HPLC (see General Information) of samples of [^18^F]**TA5** spiked with the non-radioactive reference compound **TA5** using a gradient and an isocratic mode.

### 3.5. Investigation of In Vitro Stability and Lipophilicity (logD_7.4_)

In vitro stability of [^18^F]**TA5** was studied by incubation in phosphate-buffered saline (PBS, pH 7.4), *n*-octanol and pig plasma at 37 °C for 60 min (~5 MBq of the radioligand added to 500 µL of each medium). Samples were taken at 15, 30 and 60 min and analyzed by radio-TLC and radio-HPLC (see General Information).

The lipophilicity of [^18^F]**TA5** was examined by partitioning between *n*-octanol and phosphate-buffered saline (PBS, pH 7.4) at ambient temperature using the conventional shake-flask method. The radioligand (10 µL, ~1 MBq) was added to a tube containing the *n*-octanol/PBS-mixture (6 mL, 1:1, *v*/*v*, fourfold determination). The tubes were shaken for 20 min using a mechanical shaker (HS250 basic, IKA Labortechnik GmbH & Co. KG, Staufen, Germany) followed by centrifugation (5000 rpm for 5 min) and separation of the phases. Aliquots were taken from the organic and the aqueous phase (1 mL each) and activity was measured with an automated gamma counter (1480 WIZARD, Fa. Perkin Elmer, Waltham, MA, USA). The distribution coefficient (D) was calculated as [activity (cpm/mL) in *n*-octanol]/[activity (cpm/mL) in PBS, pH 7.4] stated as the decade logarithm (logD_7.4_).

### 3.6. Animal Studies

All animal procedures were approved by the Animal Care and Use Committee of Saxony (TVV 08/13).

#### 3.6.1. In Vitro Autoradiographic Studies

Cryosections of brains obtained from juvenile female German landrace pigs (10–13 kg) and female SPRD rats (10–12 weeks old) were thawed, dried in a stream of cold air, and preincubated for 10 min with incubation buffer (50 mM TRIS-HCl, pH 7.4, 120 mM NaCl, 5 mM KCl, 2 mM CaCl_2_, 5 mM MgCl_2_) at ambient temperature.

Brain sections were incubated with ~1 MBq/mL of [^18^F]**TA5** in incubation buffer for 60 min at ambient temperature. Afterwards sections were washed twice with 50 mM TRIS-HCl (pH 7.4) for 2 min at 4 °C, dipped briefly in ice-cold deionized water, dried in a stream of cold air and exposed for 60 min to an ^18^F-sensitive image plate that was analyzed afterwards using an image plate scanner (HD-CR 35; Duerr NDT GmbH, Bietigheim Bissingen, Germany). Non-specific binding of the radioligand was determined by co-incubation with 10 µM **TA1** or **TA5**.

#### 3.6.2. In Vivo Metabolism Studies

The radioligand [^18^F]**TA5** (~70 MBq in 150 µL isotonic saline) was injected in female CD-1 mice (10–12 weeks old) via the tail vein. Brain and blood samples were obtained at 30 min p.i., plasma separated by centrifugation (14,000× *g*, 1 min), and brain homogenized in ~1 mL isotonic saline on ice (10 strokes of a PTFE plunge at 1000 rpm in a borosilicate glass cylinder; Potter S Homogenizer, B. Braun Melsungen AG, Melsungen, Germany).

#### Conventional Extraction Method

The twofold extractions were performed as a double determination. Plasma (2 × 50 µL) and brain samples (2 × 250 µL) were added each to an ice-cold acetone/water mixture (4:1, *v*/*v*; plasma or brain sample/organic solvent, 1:4, *v*/*v*). The samples were vortexed for 1 min, incubated on ice for 10 min (first extraction) or 5 min (second extraction) and centrifuged at 10,000 rpm for 5 min. Supernatants were collected and the precipitates were re-dissolved in ice-cold acetone/water (100 µL) for the second extraction. Activity of aliquots from supernatants of each extraction step and of the precipitates was quantified using an automated gamma counter (1480 WIZARD, Fa. Perkin Elmer, Waltham, MA, USA). The combined supernatants from both extractions were concentrated at 70 °C under nitrogen stream and analyzed by radio-HPLC (see General Information). Notably, [^18^F]**TA5** was quantitatively extracted from the biological material as proven by in vitro incubation of the radioligand in pig plasma and subsequent extraction applying the same protocol as for the in vivo metabolism studies. 

#### Micellar Liquid Chromatography (MLC)

Plasma (20–50 µL) was dissolved in 100–300 µL of 200 mM aqueous SDS and injected directly into the MLC system (2000 µL sample loop; see General Information). Homogenized brain material (100–200 µL) was dissolved in 500 µL of 200 mM aqueous SDS, stirred at 75 °C for 5 min and after cooling to ambient temperature injected into the MLC system.

## 4. Conclusions

The novel PDE2A radioligand [^18^F]**TA5** proved to be not suitable for molecular imaging of PDE2A protein in the brain due to (i) non-specific binding in vitro and (ii) formation of a high fraction of brain-penetrating radiometabolites in vivo. Nevertheless, **TA5** represents the most potent PDE2A ligand out of our imidazopyridotriazine-based derivatives so far. Besides, further structural modification at the side chains of the tricyclic framework is needed to possibly improve the in vitro binding properties and the in vivo stability of upcoming compounds. Notably, the developed synthetic strategies for the selective *O*-dealkylation at either the 5´-alkoxyphenyl moiety or the 2-alkoxypyridinyl function of the lead compound **TA1** are of highly importance for our ongoing work.

## Figures and Tables

**Table 1:** IC_50_ values of the novel 2-fluoroethoxy derivative **TA5** for the inhibition of human PDE2A and human PDE10A compared to our already published data for the lead compound **TA1** and the PDE2A ligands **TA2**–**4** [13,19,20].

Ligand	IC_50_ hPDE2A	IC_50_ hPDE10A	Selectivity Ratio PDE10A/PDE2A
TA5 (2-fluoroethoxy)	3.0 nM	>1000 nM	>330
TA1 (lead)	4.5 nM	670 nM	149
TA2 (5′-fluoroethoxy)	10.4 nM	77 nM	7
TA3 (5′-fluoropropoxy)	11.4 nM	318 nM	28
TA4 (5′-fluorobutoxy)	7.3 nM	913 nM	125

## Supplementary Materials

The supplementary materials are available online at http://www.mdpi.com/1420-3049/23/3/556/s1. General Information; NMR data of compounds **TA1** (incl. HR-MS) and **TA1a**; Figures S1–S6: NMR spectra of compound **TA1** (^1^H, ^13^C, ^19^F, COSY, HSQC, HMBC); Figure S7: ^1^H-NMR spectrum of compound **TA1a**; Figures S8–S10: NMR spectra of compound **TA1b** (^1^H, ^13^C-APT, ^19^F); Figures S11–S16: NMR spectra of compound **TA5** (^1^H, ^13^C-APT, ^19^F, COSY, HSQC, HMBC); Figures S17–S22: NMR spectra of compound **TA5a** (^1^H, ^13^C-APT, ^19^F, COSY, HSQC, HMBC).
