# Supplementary material for: Investigation of an 18F-labelled Imidazopyridotriazine for Molecular Imaging of Cyclic Nucleotide Phosphodiesterase 2A
Source: Molecules. 2018 Mar 2;23(3):556. doi: 10.3390/molecules23030556 (PMC6017663; doi:10.3390/molecules23030556)
Supplement: Supplementary file 1 [file molecules-23-00556-s001.pdf]

molecules

Supplementary Materials

Investigation of an ^{18}F-labelled Imidazopyridotriazine for Molecular Imaging of Cyclic Nucleotide Phosphodiesterase 2A

Susann Schröder [1,*], Barbara Wenzel [1], Winnie Deuther-Conrad [1], Rodrigo Teodoro [1], Mathias Kranz [1], Matthias Scheunemann [1], Ute Egerland [2], Norbert Höfgen [2], Detlef Briel [3], Jörg Steinbach [1] and Peter Brust [1]

1 Department of Neuroradiopharmaceuticals, Institute of Radiopharmaceutical Cancer Research, Helmholtz-Zentrum Dresden-Rossendorf, Permoserstraße 15, Leipzig 04318, Germany;
E-Mails: b.wenzel@hzdr.de; w.deuther-conrad@hzdr.de; r.teodoro@hzdr.de; m.kranz@hzdr.de; m.scheunemann@hzdr.de; j.steinbach@hzdr.de; p.brust@hzdr.de
2 BioCrea GmbH, Meissner Str. 191, Radebeul 01445, Germany;
E-Mails: ute.egerland@outlook.de; norbert.hoefgen@dynabind.com
3 Pharmaceutical/Medicinal Chemistry, Institute of Pharmacy, Faculty of Medicine, Leipzig University, Brüderstraße 34, Leipzig 04109, Germany;
E-Mail: briel@uni-leipzig.de
* Author to whom correspondence should be addressed; E-Mail: s.schroeder@hzdr.de;
Tel.: +49-341-234-179-4631.

Table of Contents

General Information; NMR data of compounds **TA1** (incl. HR-MS) and **TA1a** **S3**

Images of NMR spectra

Figure S1. ^{1}H-NMR spectrum of **TA1** (300 MHz, DMSO-d_6) **S4**

Figure S2. ^{13}C-NMR spectrum of **TA1** (75 MHz, DMSO-d_6) **S4**

Figure S3. ^{19}F-NMR spectrum of **TA1** (282 MHz, DMSO-d_6) **S4**

Figure S4. COSY-NMR spectrum of **TA1** (DMSO-d_6) **S5**

Figure S5. HSQC-NMR spectrum of **TA1** (DMSO-d_6) **S5**

Figure S6. HMBC-NMR spectrum of **TA1** (DMSO-d_6) **S6**

Figure S7. ^{1}H-NMR spectrum of **TA1a** (300 MHz, DMSO-d_6) **S7**

Figure S8. ^{1}H-NMR spectrum of **TA1b** (400 MHz, $CDCl_3$) **S8**

Figure S9. ^{13}C-APT-NMR spectrum of **TA1b** (75 MHz, $CDCl_3$) **S8**

Figure S10. ^{19}F-NMR spectrum of **TA1b** (377 MHz, $CDCl_3$) **S8**

Figure S11. ^{1}H-NMR spectrum of **TA5** (400 MHz, DMSO-d_6) **S9**

Figure S12. ^{13}C-APT-NMR spectrum of **TA5** (75 MHz, DMSO-d_6) **S9**

Figure S13. ^{19}F-NMR spectrum of **TA5** (282 MHz, DMSO-d_6) **S9**

Figure S14. COSY-NMR spectrum of **TA5** (DMSO-d_6) **S10**

Figure S15. HSQC-NMR spectrum of **TA5** (DMSO-d_6) **S10**

Figure S16. HMBC-NMR spectrum of **TA5** (DMSO-d_6) **S11**

Figure S17. ^{1}H-NMR spectrum of **TA5a** (400 MHz, DMSO-d_6) **S12**

Figure S18. ^{13}C-APT-NMR spectrum of **TA5a** (75 MHz, DMSO-d_6) **S12**

Figure S19. ^{19}F-NMR spectrum of **TA5a** (282 MHz, DMSO-d_6) **S12**

Figure S20. COSY-NMR spectrum of **TA5a** (DMSO-d_6) **S13**

Figure S21. HSQC-NMR spectrum of **TA5a** (DMSO-d_6) **S13**

Figure S22. HMBC-NMR spectrum of **TA5a** (DMSO-d_6) **S14**

General Information

NMR spectra (^1H, ^{13}C, ^{19}F) were recorded on Mercury 300/Mercury 400 (Varian, Palo Alto, CA, USA) or Fourier 300/Avance DRX 400 Bruker (Billerica, MA, USA) instruments. The hydrogenated residue of deuteriated solvents and/or tetramethylsilane (TMS) were used as internal standards for ^1H-NMR ($CDCl_3$, δ_H = 7.26; DMSO-d_6, δ_H = 2.50) and ^{13}C-NMR ($CDCl_3$, δ_C = 77.2; DMSO-d_6, δ_C = 39.5). The chemical shifts (δ) are reported in ppm (s, singlet; d, doublet; t, triplet; q, quartet; p, pentett (quintet); h, hexett (sextet); m, multiplet) and the related coupling constants (*J*) are reported in Hz. 1D and 2D NMR spectra were processed using MestReNova software (version 12.0.0-20080, rel. 2017-09-26 002, © MestreLab Res. S.L.). High resolution mass spectra (ESI +/−) were recorded on an Impact II™ instrument (Bruker Daltonics).

Compounds TA1 and TA1a

The syntheses of the lead compound **TA1** and the regioselective 5′-*O*-debutylation of **TA1** in the presence of boron tribromide to obtain the 1-phenol **TA1a** are shown in the scheme below as reported in our previous paper [1].

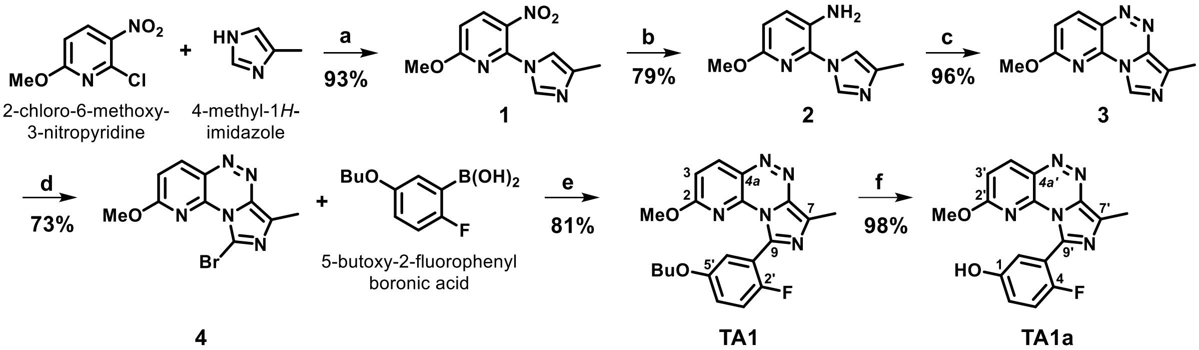

Reagents and conditions: (**a**) 3 eq TEA, DMAP (10 mol%), $CHCl_3$, 0 °C to RT, overnight; (**b**) Pd(C)/H_2, EtOH, RT, overnight; (**c**) 1.5 eq $NaNO_2$, H_2O/CH_3COOH, ≤5 °C, 30 min; (**d**) 1.5 eq *N*-bromosuccinimide (NBS), CH_2Cl_2, ≤5 °C to RT, overnight; (**e**) 1 eq 5-butoxy-2-fluorophenyl boronic acid, $[(Ph_3)P]_4Pd(0)$ (5 mol%), 3 eq K_2CO_3, 1,4-dioxane/H_2O, 90 °C, 5 h; (**f**) 3.05 eq BBr_3 (1 M in CH_2Cl_2), CH_2Cl_2, ≤5 °C, 2 h.

9-(5-Butoxy-2-fluorophenyl)-2-methoxy-7-methylimidazo[5,1-c]pyrido[2,3-e][1,2,4]triazine (**TA1**)
NMR data of compound **TA1**: ^1H-NMR (300 MHz, DMSO-d_6): δ_H = 0.90 (t, *J* = 7.4, 3H, $O(CH_2)_3C\underline{H}_3$); 1.32–1.50 (m, 2H, $O(CH_2)_2C\underline{H}_2CH_3$); 1.61–1.74 (m, 2H, $OCH_2C\underline{H}_2CH_2CH_3$); 2.79 (s, 3H, 7C-$C\underline{H}_3$); 3.46 (s, 3H, 2-$OC\underline{H}_3$); 3.98 (t, *J* = 6.4, 2H, $OC\underline{H}_2(CH_2)_2CH_3$); 7.10 (d, *J* = 8.8, $1H_{Ar}$, 3-H); 7.11 (ddd, overlap, *J* = 9.1, 4.3, 3.1, $1H_{Ar}$, 4'-H); 7.22 (dd, *J* = 5.7, 3.1, $1H_{Ar}$, 6'-H); 7.29 (t-like, *J* = 9.2, $1H_{Ar}$, 3'-H); 8.64 (d, *J* = 8.8, $1H_{Ar}$, 4-H). ^{13}C-NMR (75 MHz, DMSO-d_6): δ_C = 12.3 (s, $1C_{prim}$, 7-C-$\underline{C}H_3$); 13.6 (s, $1C_{prim}$, $O(CH_2)_3\underline{C}H_3$); 18.7 (s, $1C_{sec}$, $O(CH_2)_2\underline{C}H_2CH_3$); 30.7 (s, $1C_{sec}$, $OCH_2\underline{C}H_2CH_2CH_3$), 54.2 (s, $1C_{prim}$, 2-$O\underline{C}H_3$); 68.0 (s, $1C_{sec}$, $O\underline{C}H_2(CH_2)_2CH_3$); 112.1 (s, $1C_{ArH}$, 3-C); 115.9 (d, *J* = 23.2, $1C_{ArH}$, 3'-C); 117.3 (d, *J* = 8.3, $1C_{ArH}$, 4'-C); 117.5 (d, *J* = 1.9, $1C_{ArH}$, 6'-C); 120.2 (d, *J* = 16.3, $1C_{Ar}$, 1'-C); 127.8 (s, $1C_{Ar}$, 4a-C); 131.6 (s, $1C_{Ar}$, 9-C); 133.5 (s, $1C_{Ar}$, 10a-C); 136.6 (s, $1C_{Ar}$, 7-C); 138.9 (s, $1C_{Ar}$, 6a-C); 140.6 (s, $1C_{ArH}$, 4-C); 154.2 (d, *J* = 1.9, $1C_{Ar}$, 5'-C); 154.8 (d, overlap, *J* = 240.2, $1C_{Ar}$, 2'-C); 163.8 (s, $1C_{Ar}$, 2-C). ^{19}F-NMR (282 MHz, DMSO-d_6) δ_F = -121.98 (ddd, *J* = 9.5, 5.6, 4.3, $1F_{Ar}$, 2'-F). HR-MS (ESI) *m*/*z*: calcd. for $[C_{20}H_{21}FN_5O_2]^+$ = 382.1673; found = 382.1671 $[M+H]^+$.

4-Fluoro-3-(2-methoxy-7-methylimidazo[5,1-c]pyrido[2,3-e][1,2,4]triazin-9-yl)phenol (**TA1a**)
NMR data of compound **TA1a**: ^1H-NMR (300 MHz, DMSO-d_6) δ_H = 2.82 (s, 3H, 7'-$C\underline{H}_3$); 3.52 (s, 3H, 2'-$OC\underline{H}_3$); 6.93 (ddd, *J* = 8.9, 4.1, 3.1, $1H_{Ar}$, 6-H); 7.05 (dd, *J* = 5.8, 3.0, $1H_{Ar}$, 2-H); 7.16 (d, *J* = 8.8, $1H_{Ar}$, 3'-H); 7.20 (t, *J* = 9.2, $1H_{Ar}$, 5-H); 8.70 (d, *J* = 8.8, $1H_{Ar}$, 4'-H); 9.66 (s, 1H, 1-$O\underline{H}$).

Reference

[1] Schröder, S.; Wenzel, B.; Deuther-Conrad, W.; Teodoro, R.; Egerland, U.; Kranz, M.; Scheunemann, M.; Höfgen, N.; Steinbach, J.; Brust, P. Synthesis, ^{18}F-radiolabelling and biological characterization of novel fluoroalkylated triazine derivatives for *in vivo* imaging of phosphodiesterase 2A in brain via positron emission tomography. *Molecules* **2015**, *20*, 9591-9615.

TA1 [9-(5-Butoxy-2-fluorophenyl)-2-methoxy-7-methylimidazo[5,1-*c*]pyrido[2,3-*e*][1,2,4]triazine]

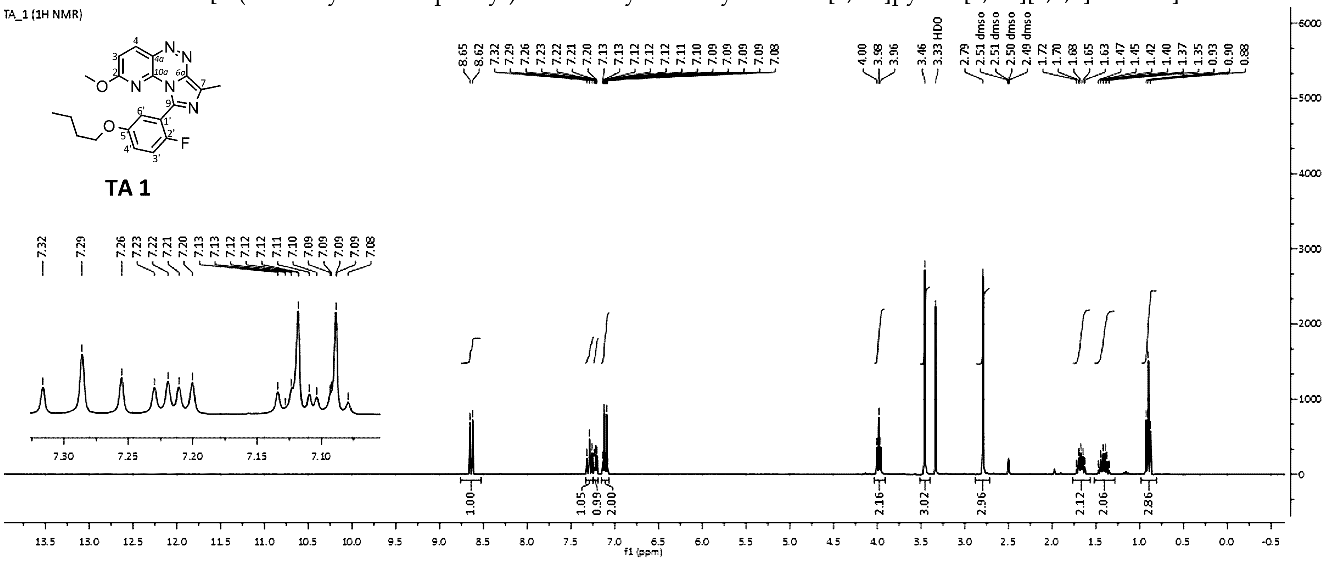

Figure S1. ^{1}H-NMR spectrum of **TA1** in DMSO-*d*$_6$.

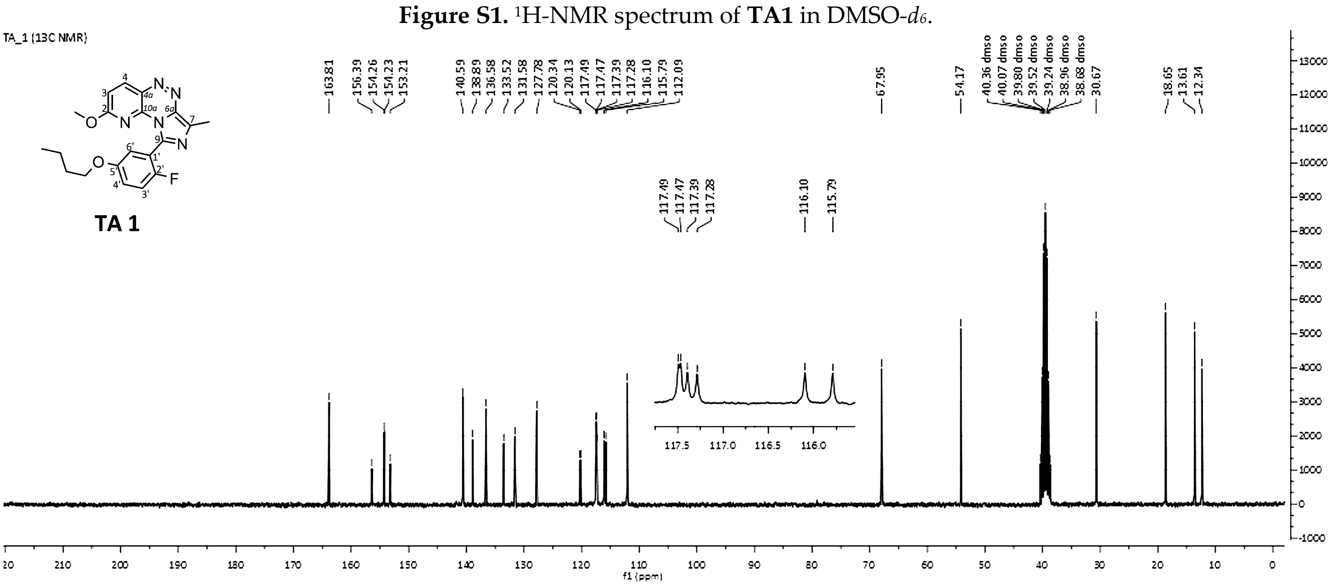

Figure S2. ^{13}C-NMR spectrum of **TA1** in DMSO-*d*$_6$.

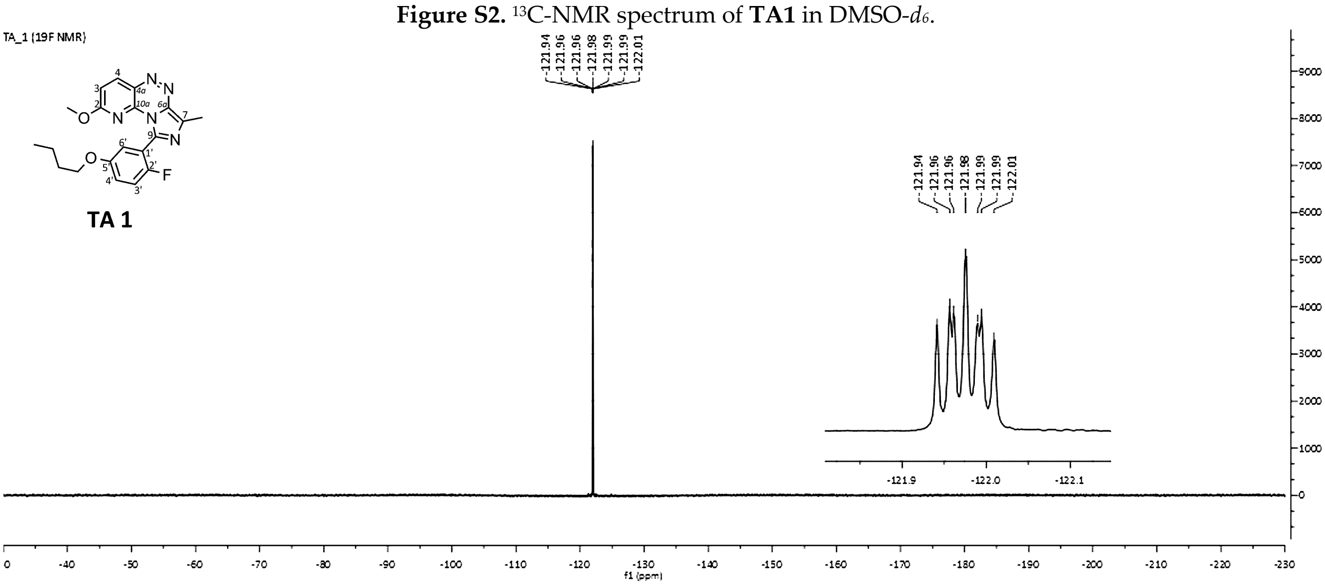

Figure S3. ^{19}F-NMR spectrum of **TA1** in DMSO-*d*$_6$.

TA1 [cont.]

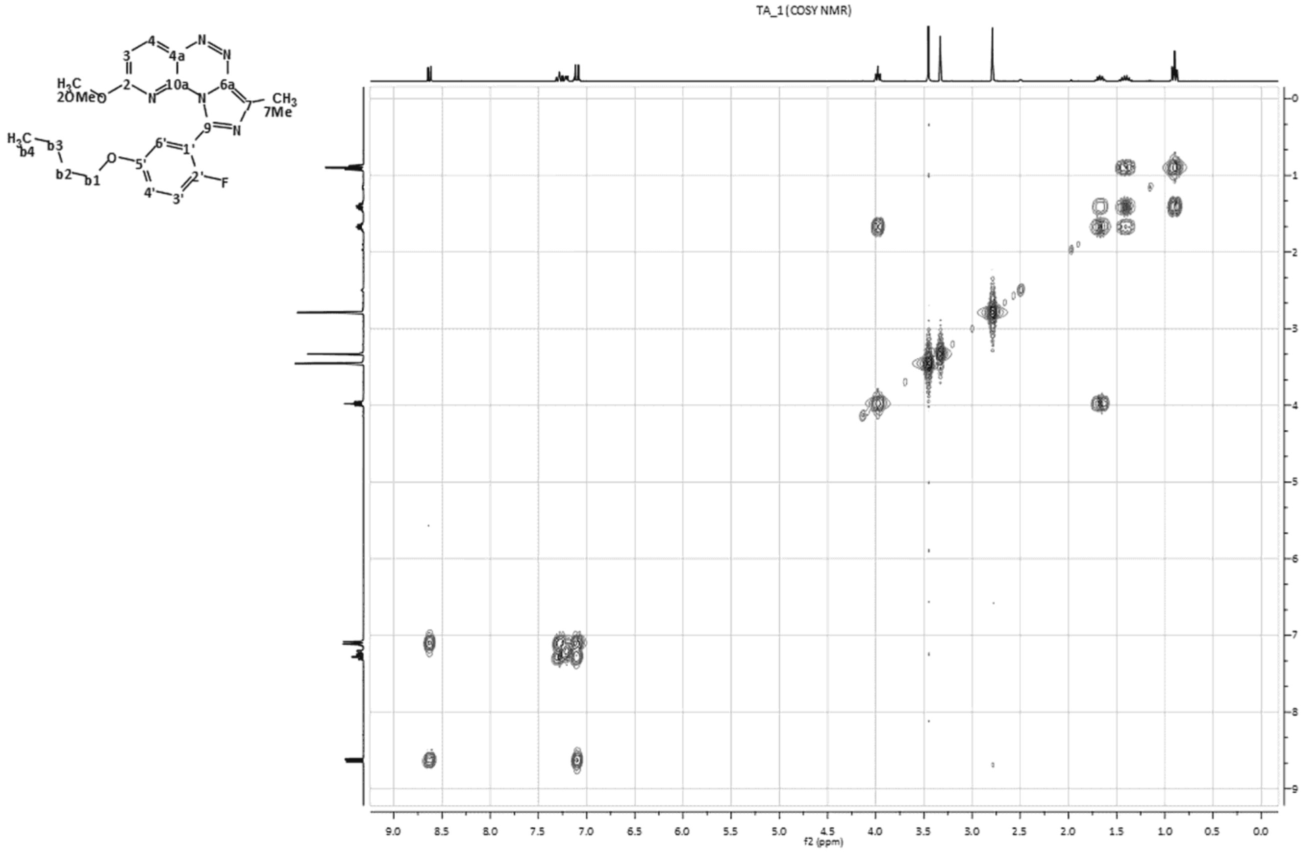

Figure S4. COSY-NMR spectrum of **TA1** in DMSO-d_6.

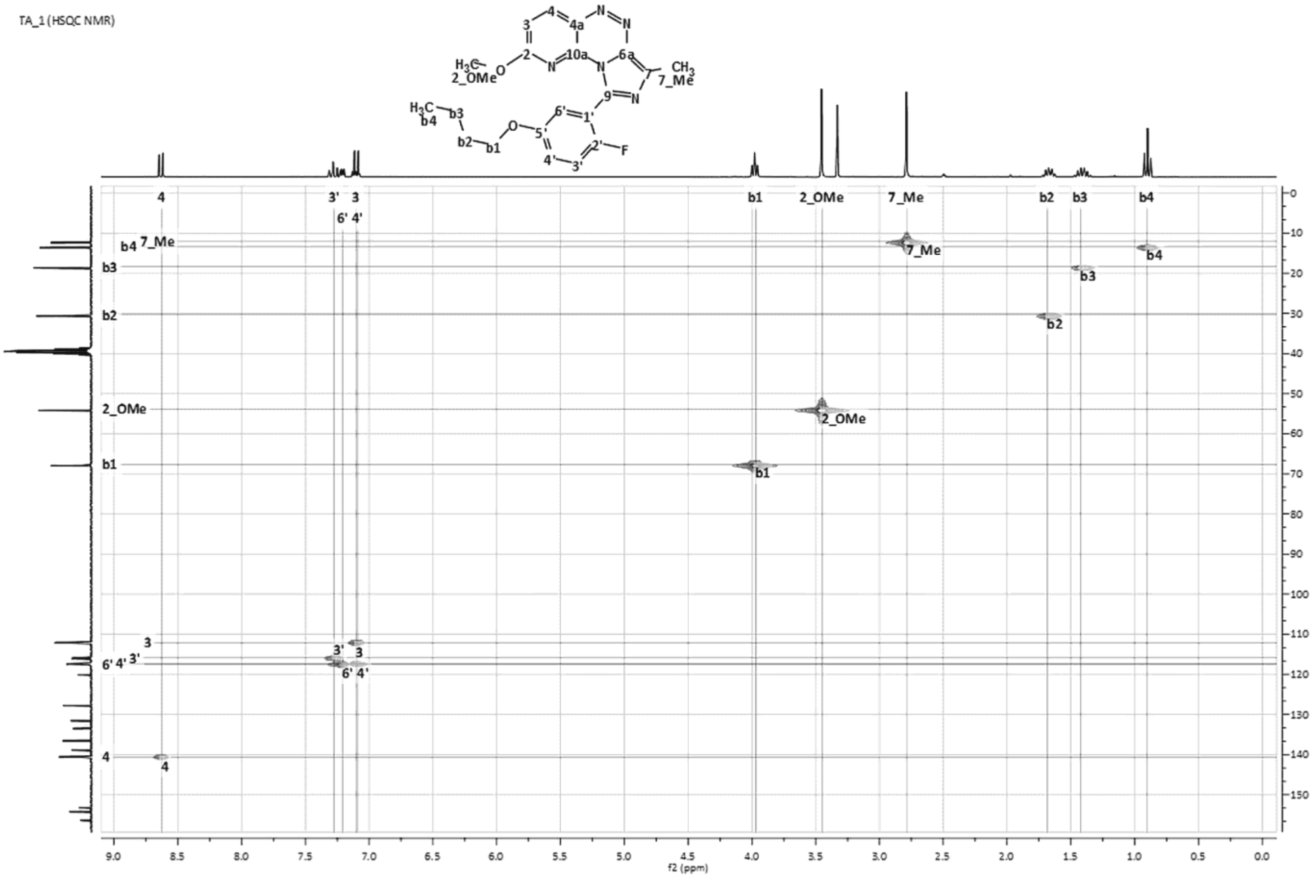

Figure S5. HSCQ-NMR spectrum of **TA1** in DMSO-d_6 (incl. assignment).

TA1 [cont.]

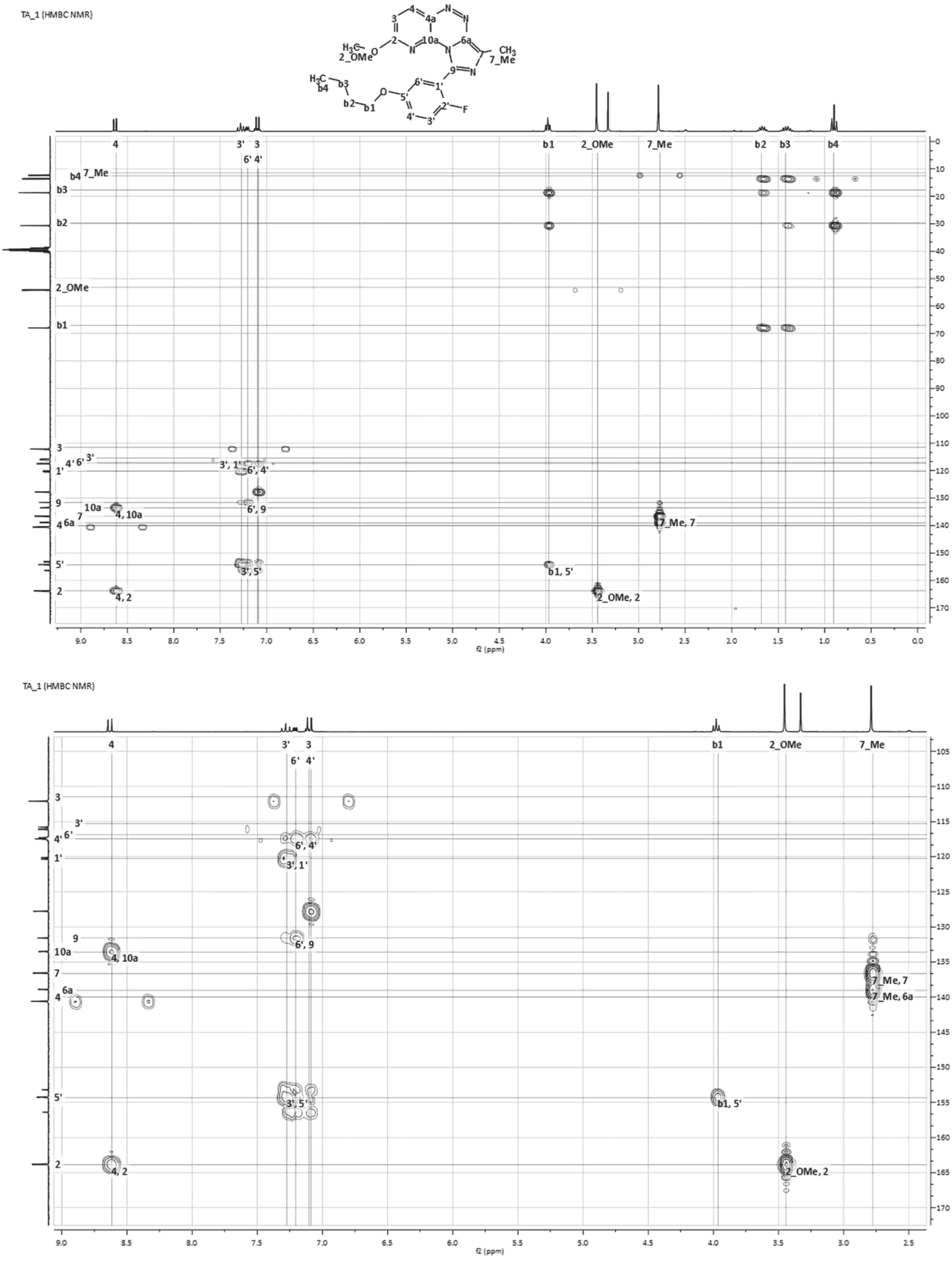

Figure S6. HMBC-NMR spectrum (top) and expansion (bottom) of **TA1** in DMSO-d_6 (incl. assignment).

TA1a [4-Fluoro-3-(2-methoxy-7-methylimidazo[5,1-*c*]pyrido[2,3-*e*][1,2,4]triazin-9-yl)phenol]

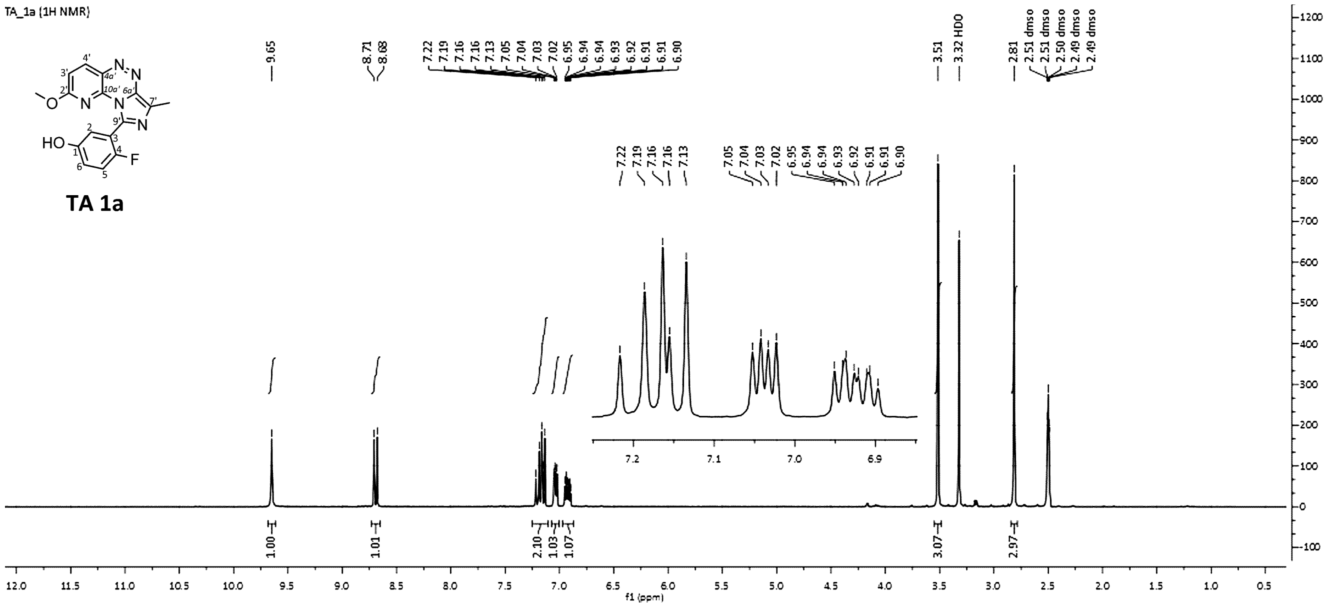

Figure S7. ^{1}H-NMR spectrum of **TA1a** in DMSO-d_6.

TA1b [9-(5-Butoxy-2-fluorophenyl)-7-methylimidazo[5,1-*c*]pyrido[2,3-*e*][1,2,4]triazin-2-ol]

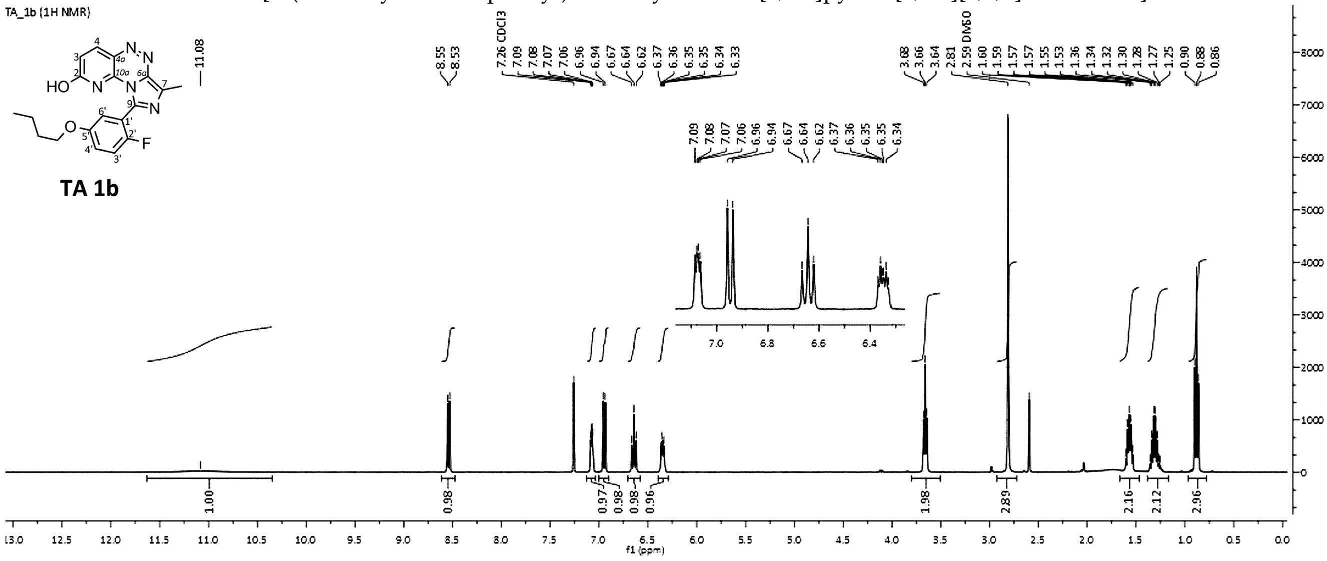

Figure S8. ^{1}H-NMR spectrum of **TA1b** in $CDCl_3$ (signal at 2.59 ppm: residual solvent of reaction [DMSO]).

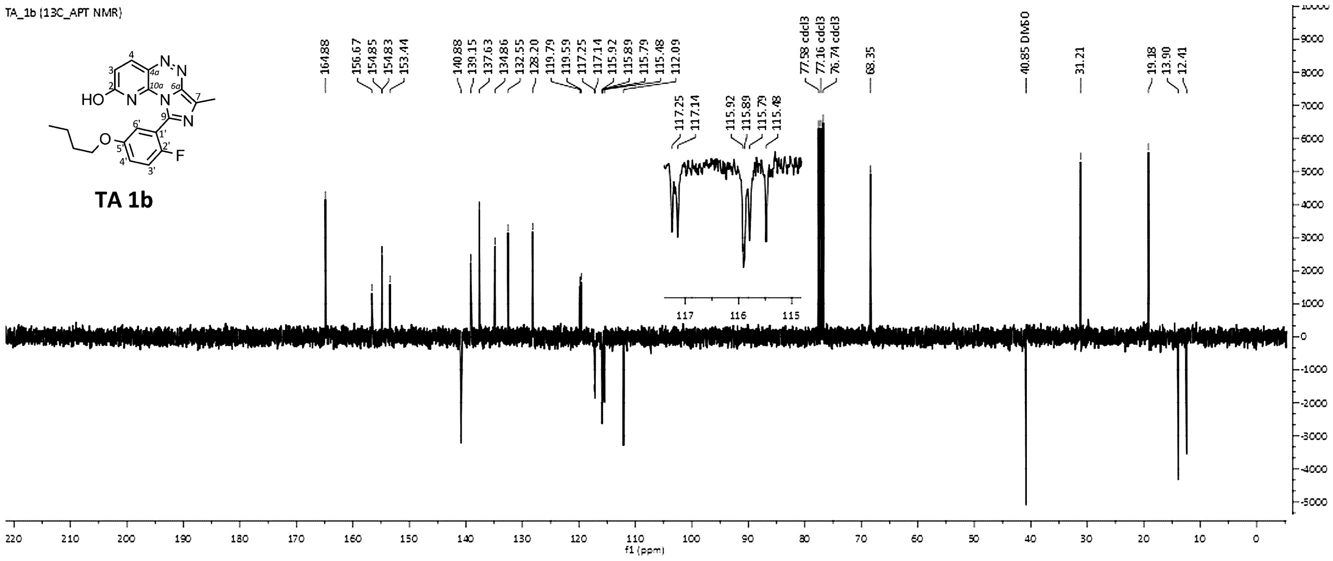

Figure S9. ^{13}C-APT-NMR spectrum of **TA1b** in $CDCl_3$ (signal at 40.85 ppm: residual solvent of reaction [DMSO]).

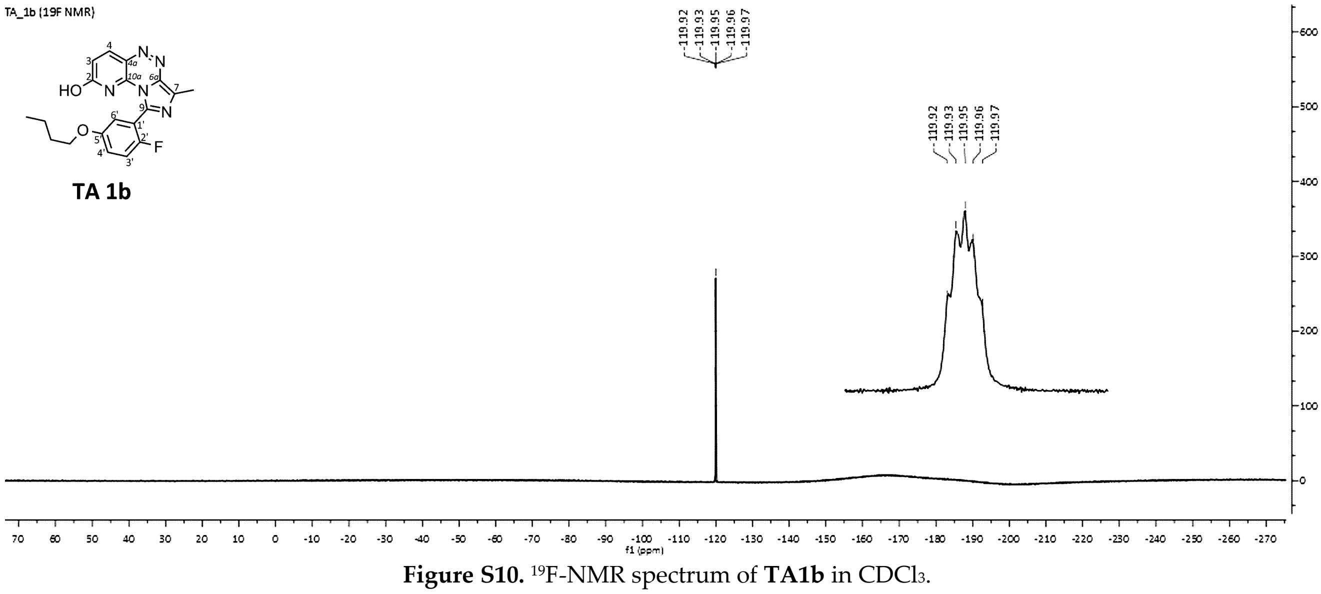

Figure S10. ^{19}F-NMR spectrum of **TA1b** in $CDCl_3$.

TA5 [9-(5-Butoxy-2-fluorophenyl)-2-(2-fluoroethoxy)-7-methylimidazo[5,1-*c*]pyrido[2,3-*e*][1,2,4]triazine]

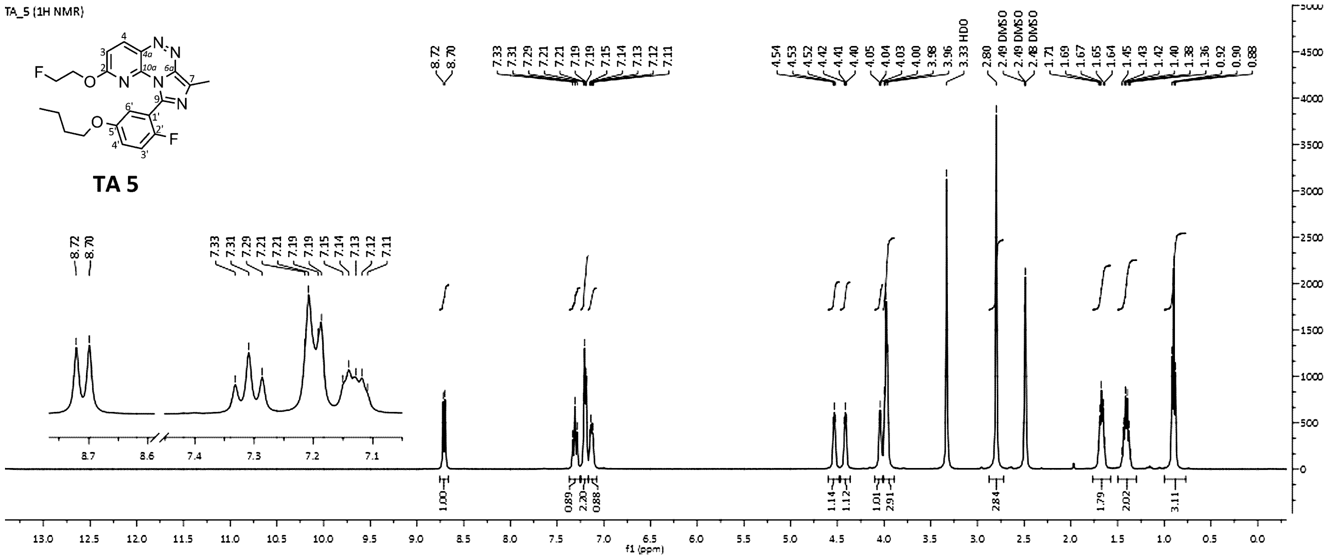

Figure S11. ^{1}H-NMR spectrum of **TA5** in DMSO-d_6.

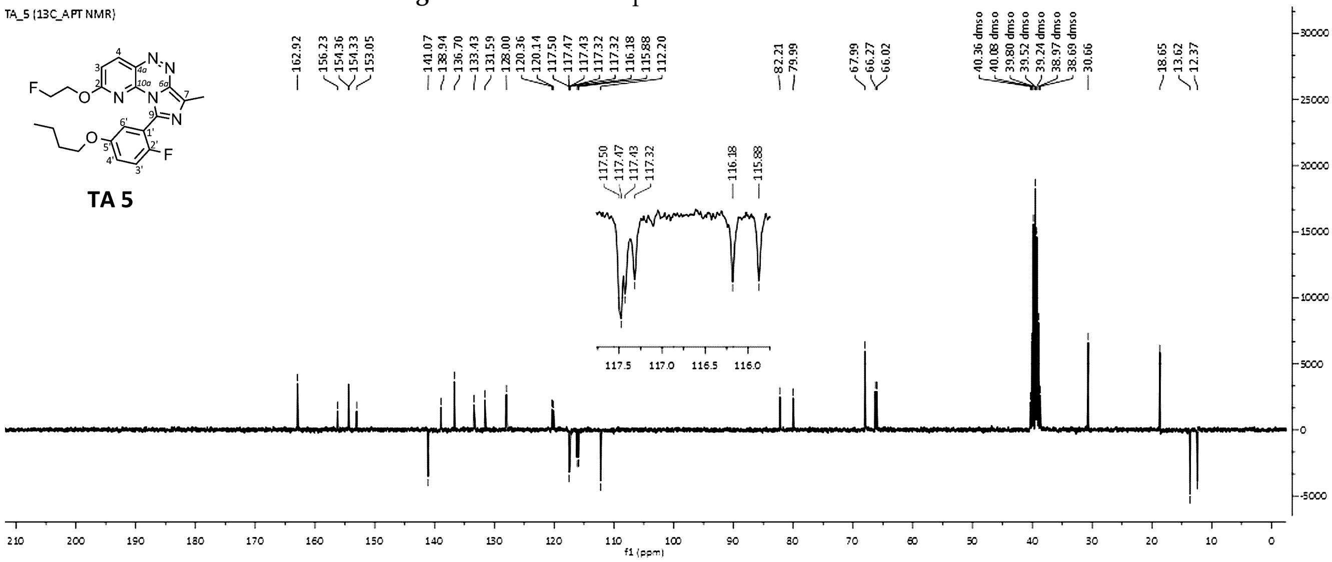

Figure S12. ^{13}C-APT-NMR spectrum of **TA5** in DMSO-d_6.

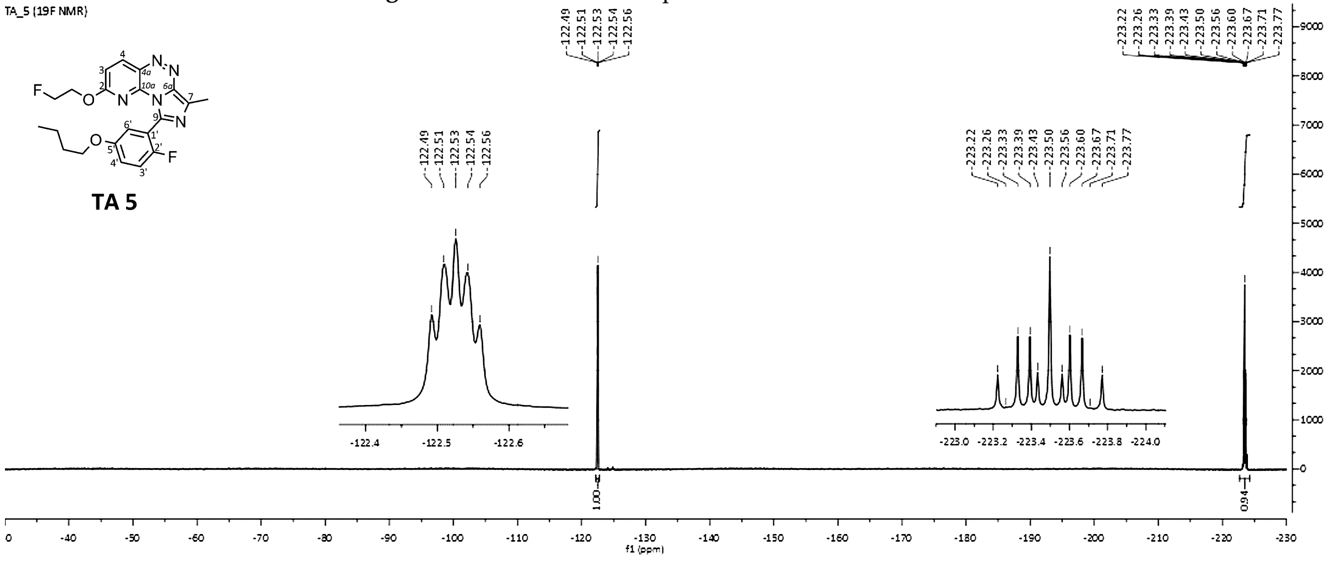

Figure S13. ^{19}F-NMR spectrum of **TA5** in DMSO-d_6.

TA5 [cont.]

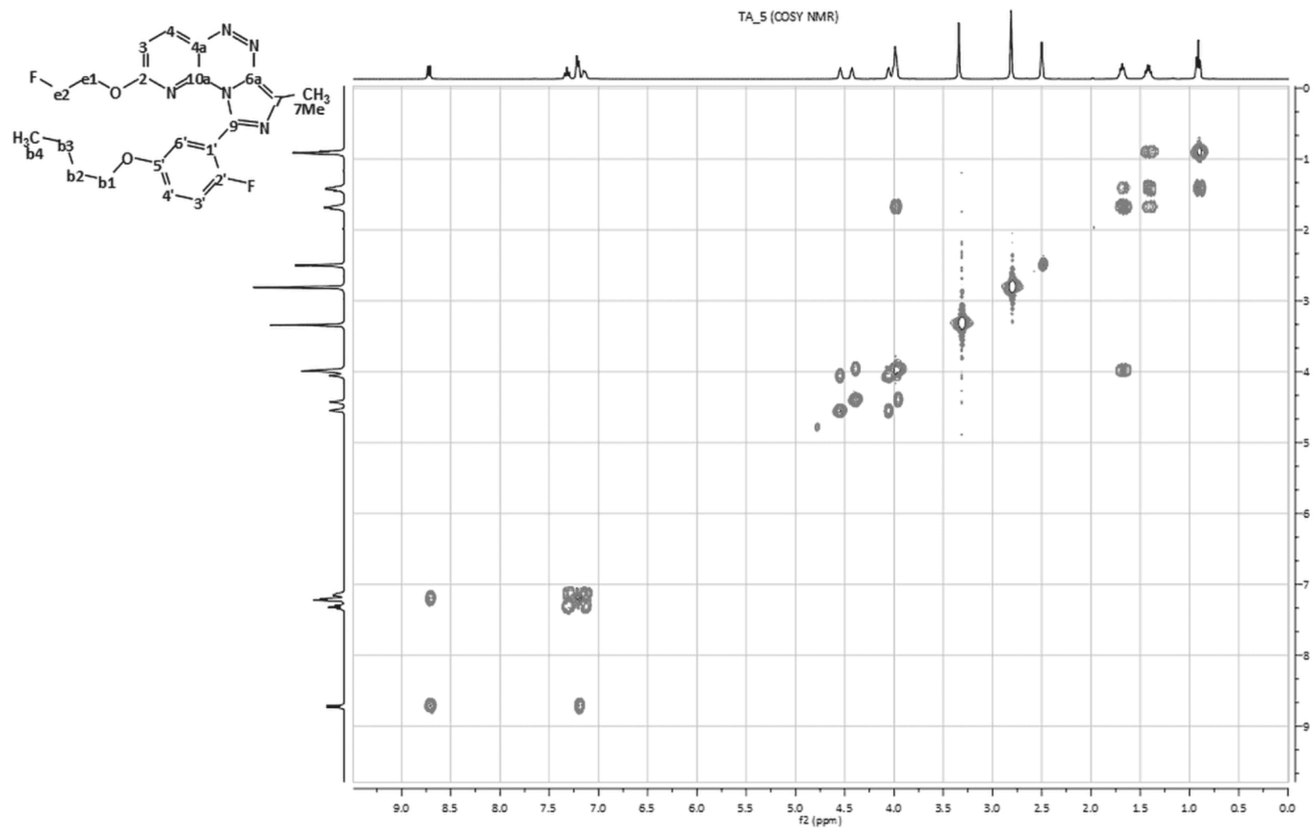

Figure S14. COSY-NMR spectrum of **TA5** in DMSO-d_6.

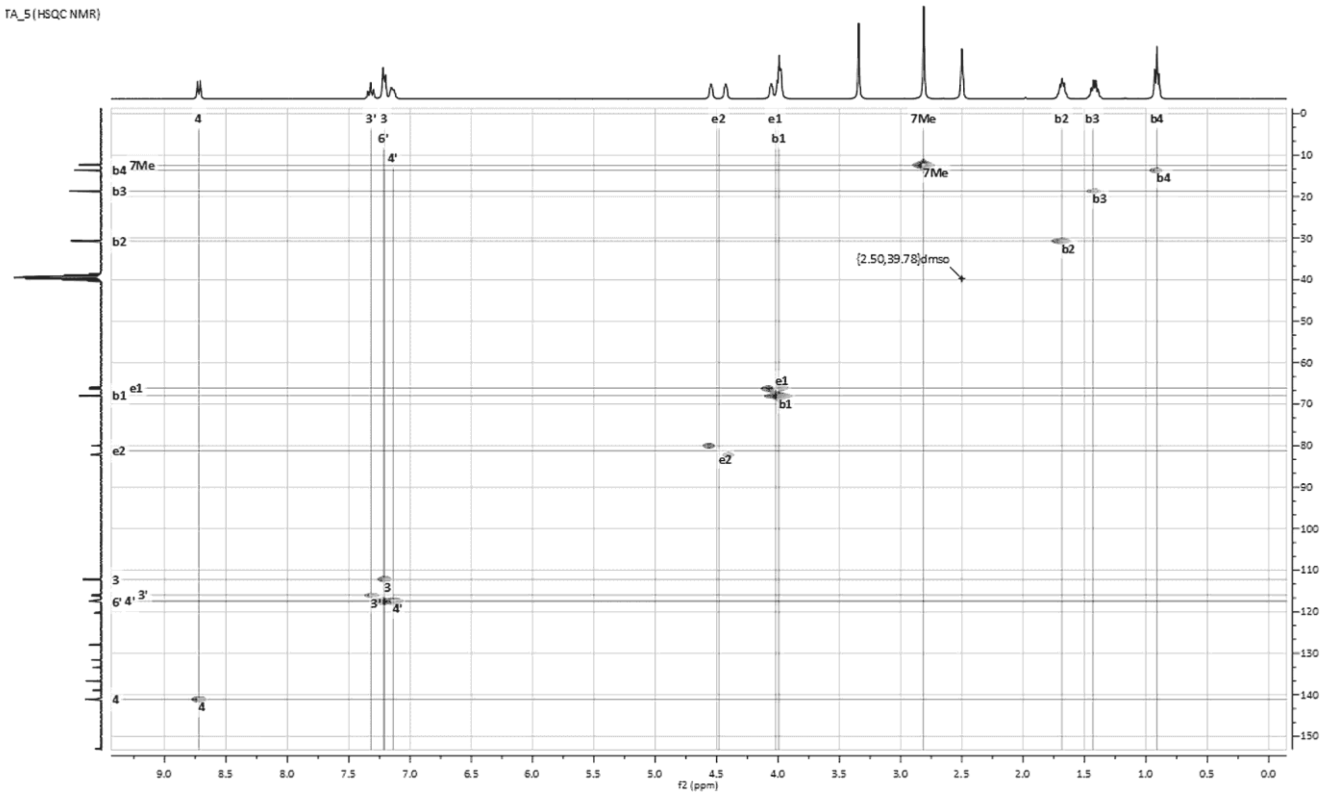

Figure S15. HSQC-NMR spectrum of **TA5** in DMSO-d_6 (incl. assignment).

TA5 [cont.]

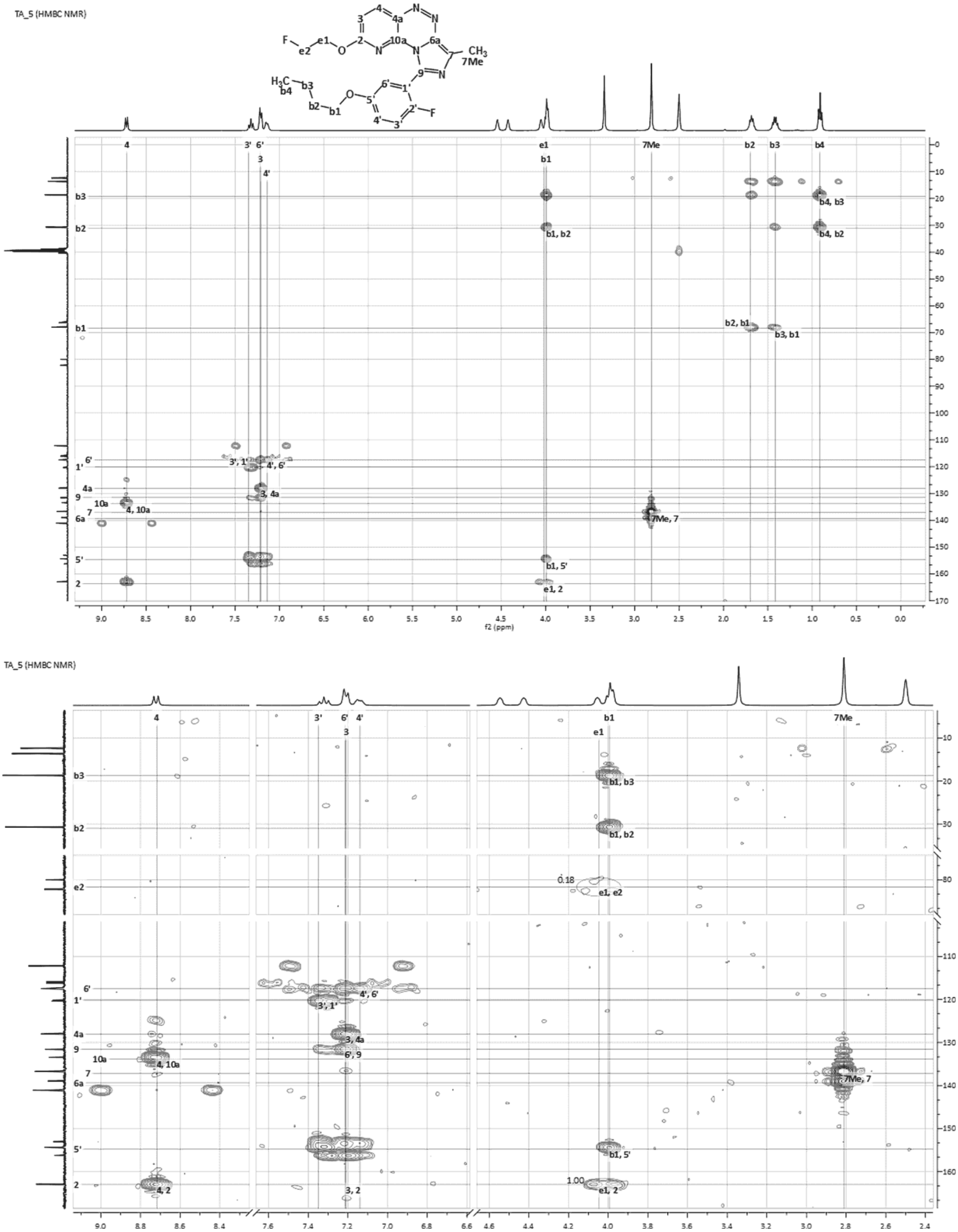

Figure S16. HMBC-NMR spectrum (top) and expansion (bottom) of **TA5** in DMSO-d_6 (incl. assignment).

TA5a [2-((9-(5-Butoxy-2-fluorophenyl)-7-methylimidazo[5,1-*c*]pyrido[2,3-*e*][1,2,4]triazin-2-yl)oxy)ethyl 4-methylbenzenesulfonate]

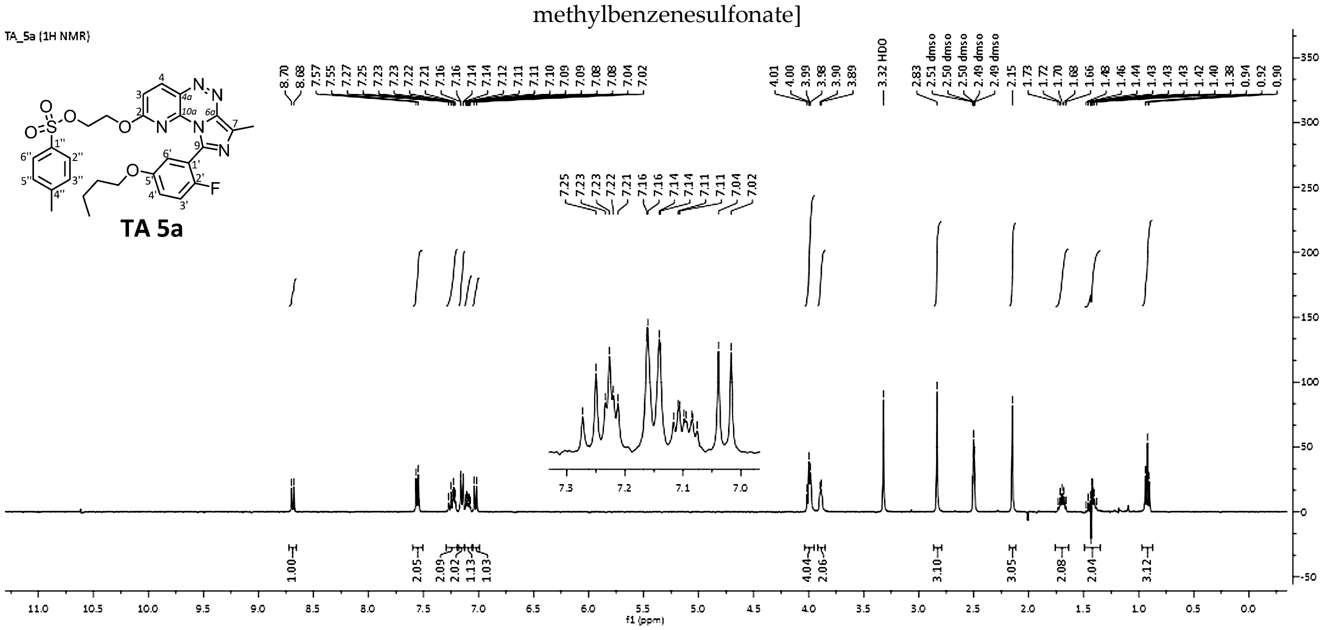

Figure S17. ^{1}H-NMR spectrum of **TA5a** in DMSO-d_6.

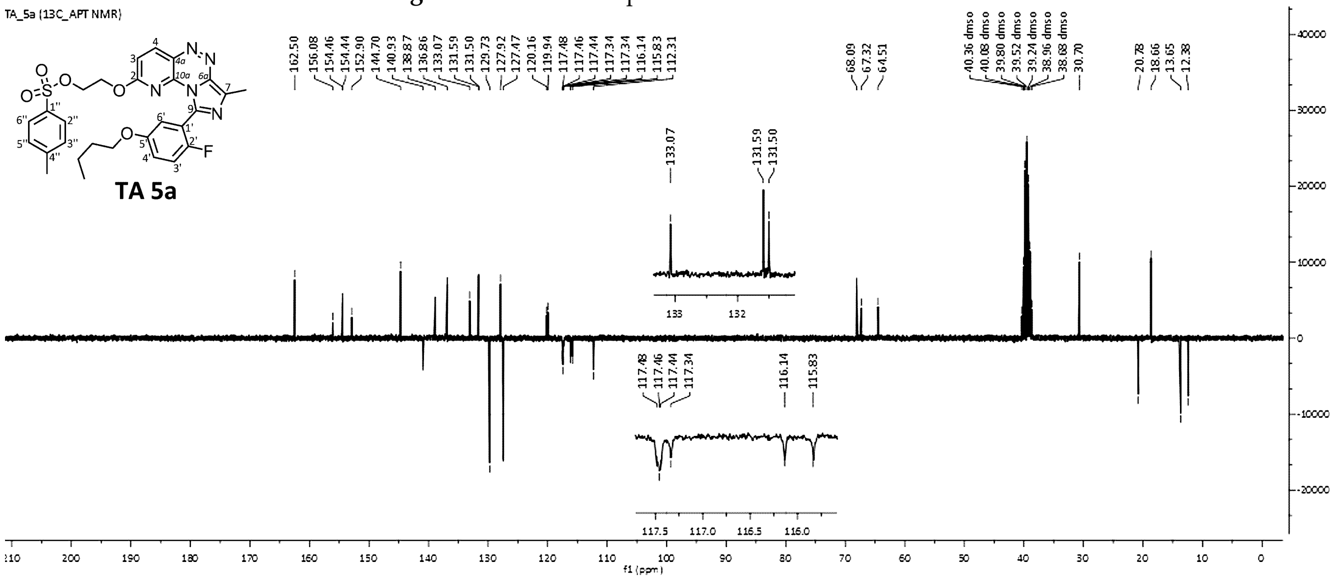

Figure S18. ^{13}C-APT-NMR spectrum of **TA5a** in DMSO-d_6.

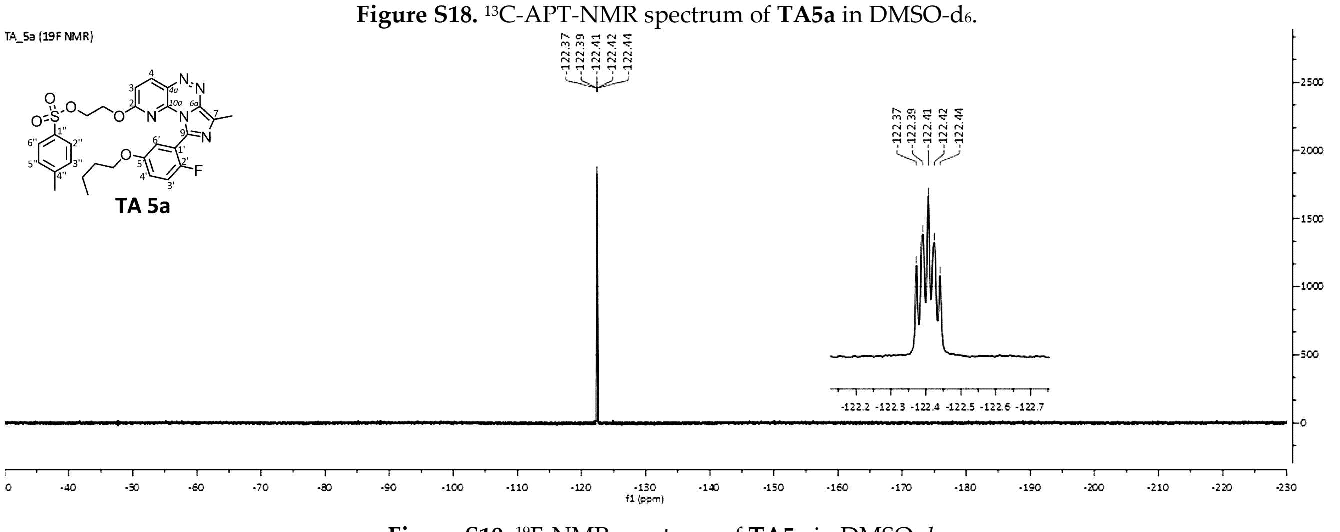

Figure S19. ^{19}F-NMR spectrum of **TA5a** in DMSO-d_6.

TA5a [cont.]

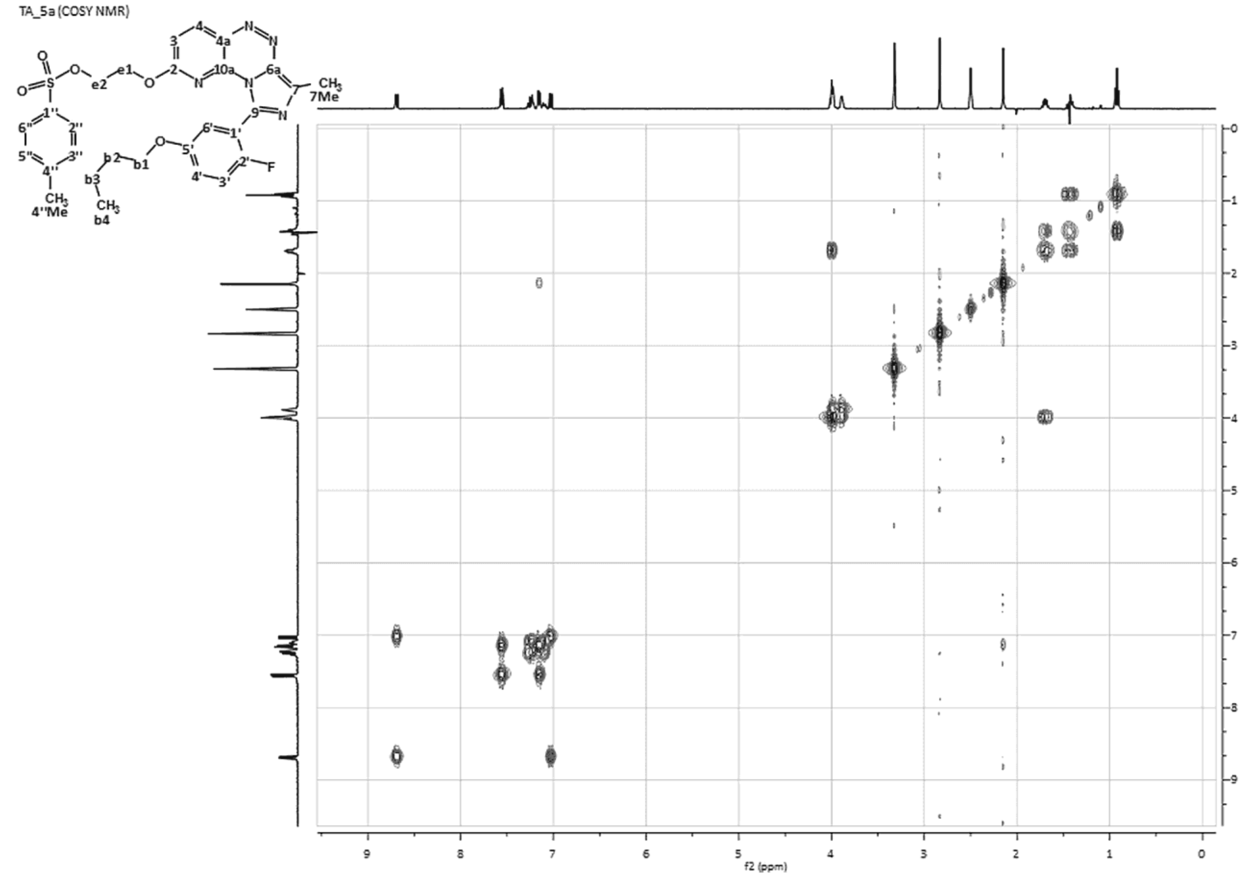

Figure S20. COSY-NMR spectrum of **TA5a** in DMSO-d_6.

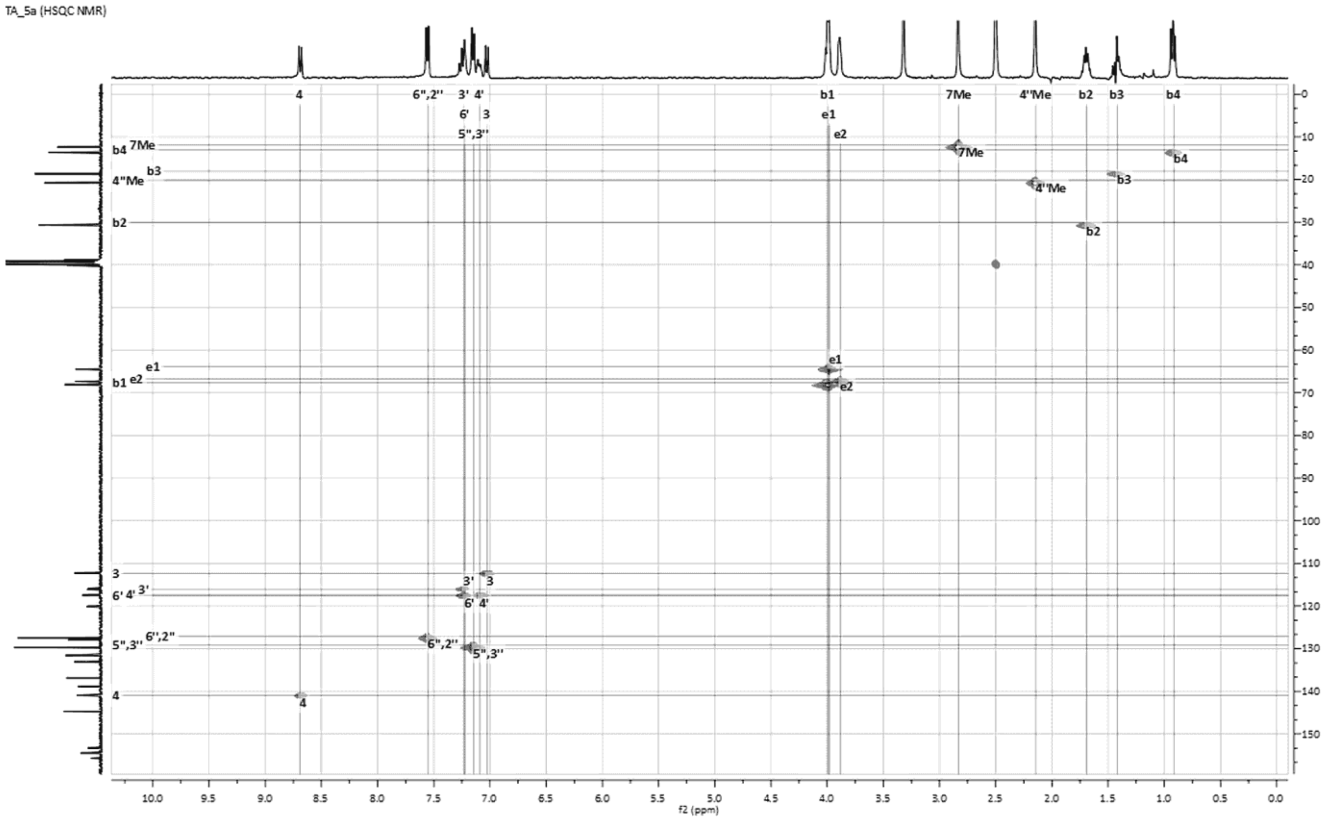

Figure S21. HSQC-NMR spectrum of **TA5a** in DMSO-d_6 (incl. assignment).

TA5a [cont.]

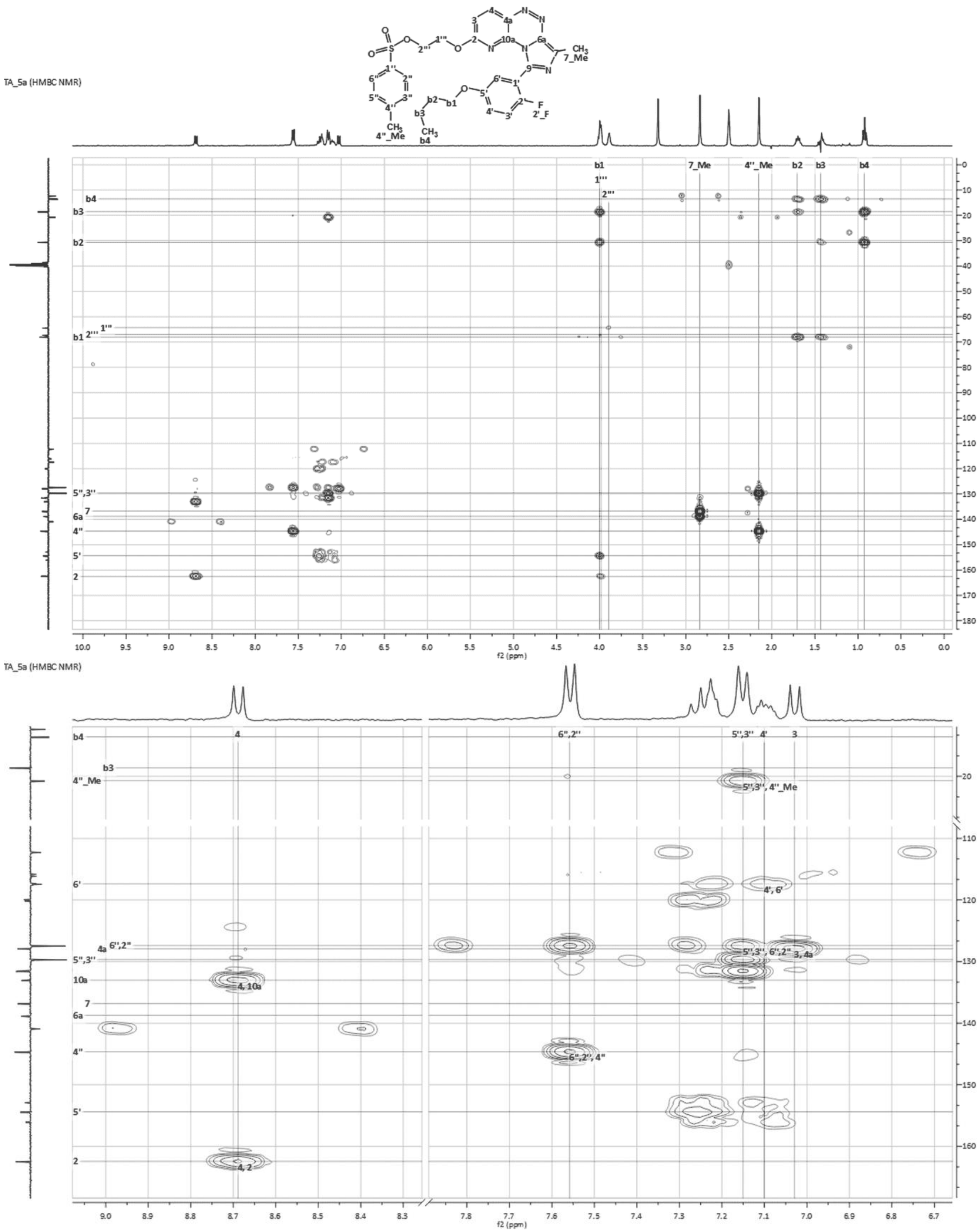

Figure S22. HMBC-NMR spectrum (top) and expansion (bottom) of **TA5a** in DMSO-d_6 (incl. assignment).
